# A systematic review on spatial crime forecasting

**DOI:** 10.1186/s40163-020-00116-7

**Published:** 2020-05-27

**Authors:** Ourania Kounadi, Alina Ristea, Adelson Araujo, Michael Leitner

**Affiliations:** 1 Department of Geoinformation Processing, University of Twente, Enschede, The Netherlands; 2 Doctoral College GIScience, Department of Geoinformatics-Z_GIS, University of Salzburg, Salzburg, Austria; 3 Boston Area Research Initiative, School of Public Policy and Urban Affairs, Northeastern University, Boston, MA USA; 4 Department of Informatics and Applied Mathematics, Federal University of Rio Grande do Norte, Natal, RN Brazil; 5 Department of Geography and Anthropology, Louisiana State University, Baton Rouge, LA USA

**Keywords:** Crime, Forecasting, Prediction, Predictive policing, Spatiotemporal, Spatial analysis, Hotspots

## Abstract

**Background:**

Predictive policing and crime analytics with a spatiotemporal focus get increasing attention among a variety of scientific communities and are already being implemented as effective policing tools. The goal of this paper is to provide an overview and evaluation of the state of the art in spatial crime forecasting focusing on study design and technical aspects.

**Methods:**

We follow the PRISMA guidelines for reporting this systematic literature review and we analyse 32 papers from 2000 to 2018 that were selected from 786 papers that entered the screening phase and a total of 193 papers that went through the eligibility phase. The eligibility phase included several criteria that were grouped into: (a) the publication type, (b) relevance to research scope, and (c) study characteristics.

**Results:**

The most predominant type of forecasting inference is the hotspots (i.e. binary classification) method. Traditional machine learning methods were mostly used, but also kernel density estimation based approaches, and less frequently point process and deep learning approaches. The top measures of evaluation performance are the Prediction Accuracy, followed by the Prediction Accuracy Index, and the F1-Score. Finally, the most common validation approach was the train-test split while other approaches include the cross-validation, the leave one out, and the rolling horizon.

**Limitations:**

Current studies often lack a clear reporting of study experiments, feature engineering procedures, and are using inconsistent terminology to address similar problems.

**Conclusions:**

There is a remarkable growth in spatial crime forecasting studies as a result of interdisciplinary technical work done by scholars of various backgrounds. These studies address the societal need to understand and combat crime as well as the law enforcement interest in almost real-time prediction.

**Implications:**

Although we identified several opportunities and strengths there are also some weaknesses and threats for which we provide suggestions. Future studies should not neglect the juxtaposition of (existing) algorithms, of which the number is constantly increasing (we enlisted 66). To allow comparison and reproducibility of studies we outline the need for a protocol or standardization of spatial forecasting approaches and suggest the reporting of a study’s key data items.

## Background

Environmental criminology provides an important theoretical foundation for exploring and understanding spatial crime distribution (Bruinsma and Johnson 2018). The occurrence of crime within an area fluctuates from place to place. Besides, crime occurrences depend on a multitude of factors, and they show an increased strategic complexity and interaction with other networks, such as institutional or community-based. In criminology research, these factors are primarily referred to as crime attractors and crime generators (Kinney et al. 2008). Spatial fluctuations and dependencies to attractors and generators suggest that crime is not random in time and in space. A strong foundation for spatial predictive crime analytics is the Crime Pattern Theory (Brantingham and Brantingham 1984). It is used to explain why crimes occur in specific areas, suggests that crime is not random, and that it can be organized or opportunistic. In particular, it shows that when the activity space of a victim intersects with the activity space of an offender, there are higher chances for a crime occurrence. The activity perimeter of a person is spatially constrained by locations that are attended (nodes). For example, if one of the personal nodes is in a high-crime neighbourhood, criminals come across new opportunities to offend.

If crime is not random it can be studied, and as such, its patterns, including the spatial component, can be modelled. As a consequence, environmental criminology theories have been tested scientifically and in the past decade various research fields have made much progress in developing methods for (spatiotemporal) crime prediction and evaluation (Caplan et al. 2011; Mohler et al. 2011, 2015; Perry 2013; Wang and Brown 2011; Yu et al. 2011).

Most prediction techniques are used for retrospective forecasting, i.e., predicting the future through historical data. Historical crime data are used alone or together with crime attractors and generators (which can be demographic, environmental, etc.) in diverse types of prediction models (Mohler et al. 2011; Ohyama and Amemiya 2018; Yu et al. 2011). Apart from static data, such as demographics or socio-economic variables, as predictors, researchers have recently included dynamic space and time features, thus giving a boost to predicting crime occurrences. These models consist of social media data (Al Boni and Gerber 2016; Gerber 2014; Kadar et al. 2017; Wang et al. 2012; Williams and Burnap 2015), and taxi pick-up and drop-off data (Kadar and Pletikosa 2018; Wang et al. 2016; Zhao and Tang 2017).

Although current crime prediction models show increasing accuracy, little emphasis has been placed on drawing the empirical and technical landscape to outline strengths and opportunities for future research, but also to identify weaknesses and threats. In this paper, we focus on spatial crime forecasting, which is the spatial forecasting of crime-related information. It has many applications such as the spatial forecast of the number of crimes, the type of criminal activity, the next location of a crime in a series, or other crime-related information. At this point, we should note that we came across papers that claim to do spatial crime forecasting or crime forecasting while extrapolating in space or detecting spatial clusters. Overall, papers in the field of spatial crime analysis use the term prediction synonymous with forecasting and they have a preference for the term prediction (Perry 2013). However, there are several spatial prediction types with applications of interpolation or extrapolation. Forecasting is a prediction that extrapolates an estimated variable into a future time. While prediction can be synonymous with forecasting, it is often also used to infer unknown values regardless of the time dimension (e.g., predict the crime in area A using a model derived from area B). Cressie (1993, pp 105–106) refers to spatial prediction as an inference process to predict values at unknown locations from data observed at known locations. His terminology includes the temporal notions of smoothing or interpolation, filtering, and prediction, which traditionally use time units instead of locations. As a result, when searching for forecasting literature you need to add the “prediction” term, which derives a much larger pool of papers, than the ones that actually do “only” forecasting. In this paper, we define the term “Spatial Crime Forecasting” as an inference approach about crime both in time and in space. In the box below, we add definition boundaries by describing variations of forecasting approaches that we consider in our study.

**Table:** 

*All forecasting approaches follow this principle:*
D_t_ (i.e., crime data in time t) is modelled to derive E_t+1_ (i.e., estimated crime information in time t + 1) that is evaluated with D_t+1_ (i.e., crime information in time t + 1).
*This principle can be applied by four forecasting approaches:*
1. D_t_ is modelled to derive E_t+1_ that is evaluated with D_t+1_.
2. D_t_ and V_static_ are modelled to derive E_t+1_ that is evaluated with D_t+1_. Where V_static_ is an explanatory variable or variables that do not change between t and t + 1.
3. D_t_ and V_dynamic_lag_ are modelled to derive E_t+1_ that is evaluated with D_t+1_. Where V_dynamic_lag_ is an explanatory variable or variables that change between t and t + 1 and lag is a period of time earlier than the time of the dependent variable.
4. D_t_, V_static_, and V_dynamic_lag_ are modelled to derive E_t+1_ that is evaluated with D_t+1_.

We are driven by the need to harmonize existing concepts and methodologies within and between criminology, sociology, geography, and computer science communities. The goal of this paper is to conduct a systematic literature review in spatial crime predictive analytics, with a focus on crime forecasting, to understand and evaluate the state of the art concerning concepts and methods given the unprecedented pace of published empirical studies. Below, we list the research questions of this study.

1. What are the types of forecasted information for which space plays a significant role? (“Overview of selected publications on spatial crime forecasting” section).
2. What are the commonly used forecasting methods? (“Spatial crime forecasting methods” section).
3. Which are the technical similarities and differences between spatial crime forecasting models? (“Spatial crime forecasting methods” section).
4. How is predictive performance being measured in spatial crime forecasting? (“Considerations when analysing forecasting performance” section).
5. What are the commonly used model validation strategies? (“Considerations when analysing forecasting performance” section).
6. What are the main dependencies and limitations of crime forecasting performance? (“Considerations when analysing forecasting performance” section).

Before presenting the results (“Results” section) and discuss them in the form of a SWOT analysis (“Discussion” section), we summarize previous literature work on crime prediction and analytics (“Related work” section) and then present the methodology to select the papers and ensure the study quality (“Methods” section). Last, in “Conclusion” section we conclude with the main findings of each research question. With our work, we aim to shed light on future research directions and indicate pitfalls to consider when performing spatial crime forecasting.

## Related work

The papers identified as review or related-work studies (a total of 13) date back to 2003 and are connected to the keyword strategy that we used (find further details in “Study selection” section). In addition, to review papers (a total of 9), we also include two editorials, one book chapter, and one research paper, because they contain an extensive literature review in the field of crime predictive analytics.

Five papers focus on data mining with a much broader scope than our topics of interest, i.e., prediction, forecasting, or spatial analysis. The oldest one proposes a framework for crime data mining (Chen et al. 2004). It groups mining techniques into eight categories, including (a) the *entity extraction* (usage example: to identify persons), (b) *clustering* (usage example: to distinguish among groups belonging to different gangs), (c) *association rule mining* (usage example: to detect network attacks), (d) *sequential pattern mining* (usage example: same as before), (e) *deviation detection* (usage example: to identify fraud), (f) *classification* (usage example: to identify e-mail spamming), (g) *string comparator* (usage example: to detect deceptive information), and (h) *social network analysis* (usage example: to construct the criminal’s role in a network). Association rule, clustering, and classification are the ones that have been discussed in other crime data mining reviews, such as for the identification of criminals (i.e., profiling) (Chauhan and Sehgal 2017), applications to solve crimes (Thongsatapornwatana 2016), and applications of criminal career analysis, investigative profiling, and pattern analysis (with respect to criminal behaviour) (Thongtae and Srisuk 2008). Furthermore, Hassani et al. (2016) conducted a recent in-depth review that looked at over 100 applications of crime data mining. Their taxonomy of applications identifies five types that include those previously described by Chen et al. (2004) with the exemption of sequential pattern mining, deviation detection, and string comparator. Regarding specific algorithms, the emphasis is put on three types, namely decision trees, neural networks, and support vector machines. Chen et al. (2004) work covers a broad spectrum of crime analysis and investigation and as such, it identifies a couple of studies related to hotspot detection and forecasting under the mining categories of clustering and classification. These technical review studies gave us examples of the data items that we need to extract and analyse, such as the techniques that are used and the tasks that are performed (Thongsatapornwatana 2016) as well as the study purpose and region (Hassani et al. 2016).

The oldest, yet still relevant paper to our work is an editorial to six crime forecasting studies (Gorr and Harries 2003). The authors refer to crime forecasting as a new application domain, which includes the use of geographical information systems (GIS), performs long- and short-term prediction with univariate and multivariate methods, and fixed boundary versus ad hoc areal units for space and time-series data. More than 15 years later, this application domain is not new but it still involves the same characteristics as described above. Another editorial by Kennedy and Dugato (2018) introduces a special issue on spatial crime forecasting using the Risk Terrain Modelling (RTM) approach. The focus of most papers is to analyse factors that lead to accurate forecasts because the foundation of the RTM approach is based on the Theory of Risky Places by Kennedy and Caplan (2012). This theory starts with the proposition that places vary in terms of risk due to the spatial influence of criminogenic factors. Last, a recent review study summarizes past crime forecasting studies of four methods, namely support vector machines, artificial neural networks, fuzzy theory, and multivariate time series (Shamsuddin et al. 2017). The authors suggest that researchers propose hybrid methods to produce better results. In our study we group and discuss a much wider number of methods (a list of 66 in Additional file 1 C) and we also identified hybrid approaches (e.g., ensemble methods) one of which dates back to 2011.

In addition, we identified two papers that describe spatial methods for spatial crime prediction per se. The paper by Bernasco and Elffers (2010) discusses statistical and spatial methods to analyse crime. They interestingly distinguish two types of spatial outcomes for modelling, including spatial distribution and movement. When it comes to spatial distribution, which is relevant to the scope of our paper, the authors describe the following spatial methods, including spatial regression models, spatial filtering, geographically weighted regression, and multilevel regression with spatial dependence. The paper by Chainey et al. (2008) focuses on hotspot mapping as a basic approach to crime prediction. The techniques they describe and empirically examine are spatial ellipses, thematic mapping of geographic areas, grid thematic mapping, and Kernel Density Estimation (KDE). Among these, KDE yielded the highest prediction accuracy index (PAI) score. Surprisingly, most of the spatial methods (with the exemption of KDE and RTM) have not been used by authors of our selected papers (see methods discussed in “Spatial crime forecasting methods” section).

Regarding predictive policing, a recent review explains its definition, how it works, how to evaluate its effectiveness, and it also provides an overview of existing (mostly commercial) applications (Hardyns and Rummens 2018). One of the innovative aspects of this review is the section on the evaluation of predictive policing using three criteria, namely the correctness of the prediction, the effect of predictive policing implementations to actual crime rates, and the costs relative to the methods being replaced. The authors of this paper support the definition of predictive policing that originates from Ratcliffe (2015, p. 4), which reads: “*the use of historical data to create a spatiotemporal forecast of areas of criminality or crime hot spots that will be the basis for police resource allocation decisions with the expectation that having officers at the proposed place and time will deter or detect criminal activity*”. In general, spatial crime forecasting has a broader scope and is not synonymous to predictive policing. In addition, the papers that we examine do not aim in assisting policing decisions (although this can be an indirect consequence) but they have an academic and explanatory focus. However, the effectiveness of the predictive analysis- first criterion, as framed by Hardyns and Rummens (2018), is highly connected to our scope and thus is further analysed, from a technical perspective, in “Considerations when analysing forecasting performance” section.

A second predictive policing systematic review by Seele (2017) examines the potential of big data to promote sustainability and reduce harm and also discusses ethical and legal aspects linked to predictive algorithms. Similarly, Ozkan (2018) also reviews big data for crime research. This paper provides a critical discussion on the benefits and limitations of data-driven research and draws attention to the imminent threat of eliminating conventional hypothesis testing, which has traditionally been an integral scientific tool for social scientists and criminologists.

Except for Seele (2017) no other related-work study follows a systematic procedure regarding the methods to identify and select relevant research, and thereafter to collect and analyse data from them. Also, our work focuses only on spatial crime forecasting, which is narrower than crime data mining and broader than predictive policing as discussed above. Last, we aim to contribute with scientific reference for technical issues in future studies. To achieve this, we follow a review protocol (“Methods” section), to answer six research questions (mentioned in “Background”) that have not been systematically addressed by previous studies.

## Methods

### Study selection

**Fig. 1:**
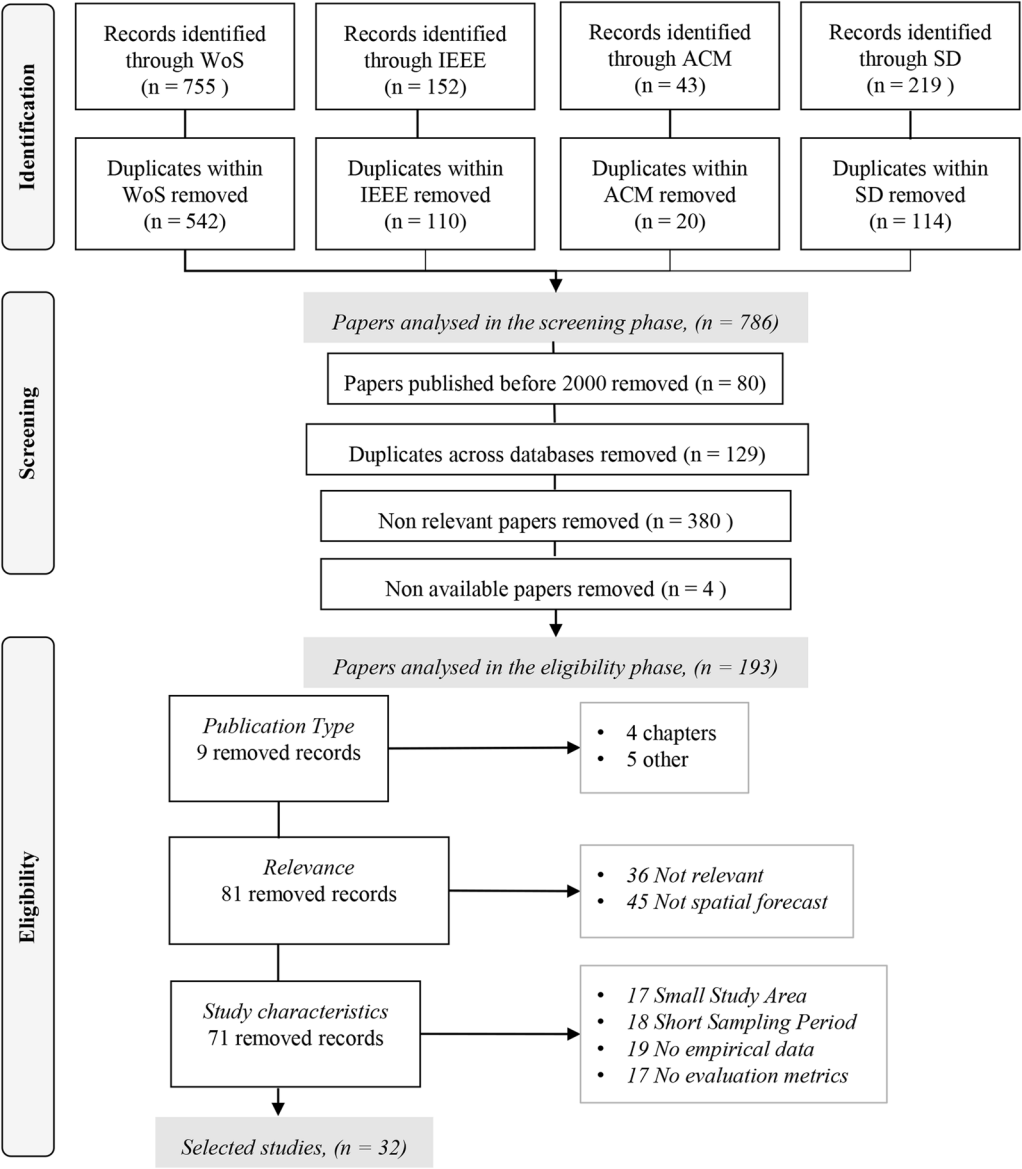
The three phases of the study selection process: identification, screening, and eligibility

This study follows the reporting guidance “PRISMA” (Preferred Reporting Items for Systematic reviews and Meta-Analyses) (Liberati et al. 2009). PRISMA suggests a checklist of 27 items regarding the sections of a systematic literature review and their content, as well as a four-phase flow diagram for the selection of papers. We adopted and modified the PRISMA guidance according to the needs of our study. Our flow diagram contains three phases for the selection of papers. The first phase is “identification” and involves the selection of information sources and a search strategy that yields a set of possible papers. The second phase is “screening” the selected papers from the first phase, and removing the ones that are not relevant to the research scope. The third phase is “eligibility”, in which we applied a more thorough reading of papers and selected the ones that are relevant to our research questions. The count of papers in each phase and their subsequent steps are illustrated in Fig. 1.

The number of papers selected in the Identification phase is based on eleven keywords related to crime prediction (i.e., predict crime, crime predictive, predictive policing, predicting crimes, crime prediction, crime forecasting, crime data mining, crime mining, crime estimation, crime machine learning, crime big data). In addition, we added seven more spatially explicit terms (i.e., crime hotspot, spatial crime prediction, crime risk terrain modelling, spatial crime analysis, spatio-temporal modelling crime, spatiotemporal modelling crime, near-repeat crime). In a subsequent analysis, we have visualized the word frequency of the titles of the selected papers as evidence of the relevance of the keywords used. This can be found in Additional file 1 B: *A word cloud of high*-*frequency words extracted from the titles of the selected papers*.

Next, we selected information sources to perform literature searches. Although there is a remarkable number of search engines and academic databases, we focus on scholarly and comprehensive research databases including fields where spatial crime prediction is a representative topic. We considered the following databases, including Web of Science by Clarivate Analytics (WoS), Institute of Electrical and Electronics Engineers (IEEE) Xplore, ScienceDirect by Elsevier (SD), and Association for Computing Machinery (ACM) Digital Library. We consider that an optimal search process should include multiple academic search databases, with searches being carried out at the best level of detail possible. In addition, as also discussed by Bramer et al. (2017) in an exploratory study for database combinations, if the research question is more interdisciplinary, a broader science database such as Web of Science is likely to add value. With regards to Google Scholar (GS) there are divergent opinions between researchers if GS brings relevant information for an interdisciplinary review or not. Holone (2016) discusses that some engine searches, specifically GS, have a tendency to selectively expose information by using algorithms that personalize information for the users, calling this the filter bubble effect. Haddaway et al. (2015) found that when searched for specific papers, the majority of the literature identified using Web of Science was also found using GS. However, their findings showed moderate to poor overlap in results when similar search strings were used in Web of Science and GS (10–67%), and that GS missed some important literature in five of six case studies.

In each database, we used keywords on singular and plural word versions (e.g., crime hotspot/s). For WoS, we used the advanced search option, by looking for papers written in English and matching our keywords with the topic or title. For IEEE, we searched for our keywords in the metadata or papers’ titles. In SD and ACM, we used the advanced search option with Boolean functions that searched our keywords in the title, abstract, or paper’s keywords. The identified papers were integrated directly into the free reference manager Mendeley. Last, we removed duplicates within each database, which resulted in 786 papers for the second phase, the Screening phase. The last search in the Identification phase was run on 5 February 2019.

Whereas, the use of statistical and geostatistical analyses for crime forecasting has been considered for quite some time, during the last two decades there has been an increasing interest in developing tools that use large data sets to make crime predictions (Perry 2013). Thus, predictive analytics have been included in law enforcement practices (Brayne 2017). This is the main reason that during the Screening phase, we first excluded papers published before 2000. Second, we removed duplicates across the four selected databases (WoS, IEEE, SD, and ACM). Third, we screened all papers to identify the “non-relevant” ones. This decision was made by defining “relevant” papers to contain the following three elements. The first element is that a paper addresses crime events with explicit geographic boundaries. Common examples of excluded papers are the ones dealing with the fear of crime, offenders’ characteristics, offender, victims’ characteristics, geographical profiling, journey to crime, and cyber or financial crime. The second element for a paper to be “relevant” is that it employs a forecasting algorithm and is not limited to exploratory or clustering analysis. The third element is that there is some form of spatial prediction. This means that there are predefined spatial units of analysis, such as inferencing for each census block of the study area. For the relevance elements, our strategy was the following: (a) read title and screen figures and/or maps, (b) if unsure about relevance, read abstract, (c) if still unsure about relevance, search for relevant words (e.g., geo*, location, spatial) within the document. The last step of the Screening phase was to remove relevant papers that authors did not have access to, due to subscription restrictions. The Screening phase resulted in 193 relevant papers to be considered for the third and final phase.

During this final phase, the Eligibility phase, we read the abstract and main body of all 193 papers (e.g., study area, data, methods, and results). The focus was to extract data items that compose the paper’s eligibility criteria. These are grouped into three categories, namely: (a) *publication type* which is the first data item, (b) relevance: consists of data items *relevance* and *purpose*, and (c) study characteristics: consists of data items *study area*, *sampling period*, *empirical data*, *evaluation metrics*. Next, we discuss each category and the data items it entails.

The first data item is the publication type. Literature reviews sometimes exclude conference papers because their quality is not evaluated like International Scientific Indexing (ISI) papers. However, for some disciplines, such as computer science, many conferences are considered as highly reputable publication outlets. In the Screening phase, we found a large number of papers from computer or information science scholars, hence at this stage we decided not to exclude conference papers (n = 65), but also non-ISI papers (n = 19). In total, we excluded nine papers that are book chapters or belong to other categories (e.g., editorial).

The next two “relevance” criteria (i.e., relevance and purpose) address the fit of the papers’ content to our research scope. Paper relevance was checked again during this phase. For example, some papers that appeared to be relevant in the Screening phase (i.e., a paper is about crime events, space, and forecasting), were actually found not to be relevant after reading the core part of the paper. For example, prediction was mentioned in the abstract, but what the authors implied was that prediction is a future research perspective of the analysis that was actually done in the paper. Also, we added the data item “purpose” to separate methods that model and explore relationships between the dependent and independent variables (e.g., crime attractors to burglaries) from the ones that perform a spatial forecast. The number of papers that were excluded due to these criteria amounted to 81.

Last, there are four more “study characteristics” criteria relevant to the quality and homogeneity of the selected papers. First, the study area should be equal to or greater than a city. Cities are less prone to edge effects compared to smaller administrative units within a city that share boundaries with other units (e.g., districts). In addition, the smaller the study area the more likely it is that conclusions are tailored to the study characteristics and are not scalable. Second, the timeframe of the crime sample should be equal or greater than a year to ensure that seasonality patterns were captured. These two items also increase the homogeneity of the selected studies. Yet, there are significant differences among studies that are discussed further in Results section. The last two criteria are the restriction to analysing empirical data (e.g., proof of concepts or purely methodological papers were excluded) and to use measures that evaluate the models’ performance (e.g., mean square error). The last two criteria ensure that we only analyse studies that are useful to address our research questions. The number of papers that were excluded due to the publication type, the relevance, or the study characteristics were 71. Furthermore, Fig. 1 shows the number of excluded papers for each data item (e.g., 17 papers were excluded due to insufficient size of the study area). Finally, the entire selection processes yielded 32 papers.

### Study quality

Two of the four authors of this research performed the selection of the papers to be analysed. Prior to each phase, these two authors discussed and designed the process, tested samples, and divided the workload. Then, results were merged, analysed, and discussed until both authors reached a consensus for the next phase. The same two authors crosschecked several of the results to ensure methodological consistency among them. The reading of the papers during the final phase (i.e., eligibility) was performed two times, by alternating the papers’ samples among the two authors, to ensure all eligible papers were included. In addition, in case some information on the paper’s content was unclear to the two authors, they contacted via email the corresponding authors for clarifications.

Regarding the results subsections that constitute four study stages (“Study characteristics”, “Overview of selected publications on spatial crime forecasting”, “Spatial crime forecasting methods”, and “Considerations when analysing forecasting performance” sections), one or two authors performed each and all authors contributed to extracting information and reviewing them. To extract information that is structured as data items we followed a procedure of three steps that was repeated at each stage. First, the papers were read by the authors to extract manually the data items and their values (1—extract). Data items and their values were then discussed and double-checked by the authors (2—discussion/consensus). In case information was still unclear, we contacted via email the corresponding authors for clarifications (3—consultation). This information was structured as a matrix where rows represent the papers and columns are several items of processed information (e.g., a data item is the year of publication). Table 1 shows the data items at the stage at which they were exploited. The attributes (values) of the items are discussed in “Results” section.

**Table 1:** Data items analyzed at different study stages

Study stage	Data items
Identification	Authors; year; title; data source
Screening	Relevance_1; availability
Eligibility	Publication type; empirical data; performance evaluation; spatial size; temporal size; purpose, relevance_2
Results “Study characteristics”	Year; title; discipline; journal/conference; study area country, institution
Results “Overview of selected publications on spatial crime forecasting”	Study area; scale; sampling period; months; type; sample; inference; task; spatial unit; temporal unit
Results “Spatial crime forecasting methods”	Proposed method; best proposed method; baseline method; proposed algorithm type; proposed method input, variables
Results “Considerations when analysing forecasting performance”	Evaluation metric; validation strategy

*The risk of bias in individual studies* was assessed via the scale of the study. Spatial and temporal constraints were set (already defined in the eligibility phase) to ensure that we analyse medium to large scale studies and that findings are not tied to specific locality or seasonality characteristics. Furthermore, we did not identify duplicate publications (i.e., two or more papers with the same samples and experiments) and did not identify study peculiarities, such as special and uncommon features or research topics.

Last, the *risk of bias across studies* was assessed via an online survey. We contacted the authors of the publications (in some cases we could not identify contact details) and ask them to respond to a short survey regarding the results of their paper. The introductory email defined the bias across studies as “*Bias across studies may result from non*-*publication of full studies (publication bias) and selective publication of results (i.e., selective reporting within studies) and is an important risk of bias to a systematic review and meta*-*analysis”*. Then, we explained the content of the survey that is to identify, if there are non-reported results that are considerably different from the ones in their papers. This information was assessed via two questions (for further details we added the questionnaire as a Additional file 1 of this paper). Out of the 32 papers, we received responses for 11 papers (*n *= *12,* with two authors responding to the same paper). One factor that explains the low response rate is that many authors have changed positions (papers date back to 2001) and for some we could not identify their new contact details, while for others we received several non-delivery email responses.

Regarding the responses’ results, seven authors responded that they never conducted a similar study to the one for which they were contacted for and five responded that they have conducted a similar study to the one for which they were contacted. A similar study was defined as a study in which: (a) the study design, selection of independent variables/predictors, selection of method(s), and parametrization of a method(s) are the same, and (b) data can be different. From those who performed a similar study four responded that their results were not different and one responded that their results were considerably different. However, in a follow-up explanatory answer, this author responded that changing the parametrization yielded different results regarding the performance ranking of three algorithms and that the data and the study area were the same. Based on this small-scale survey there is no indication that there is a risk of bias across studies. However, further investigation of this matter is needed.

## Results

### Study characteristics

In this section, we discuss generic characteristics of the selected papers. To start with, the type of publication is slightly higher for ISI journal articles (n = 18) than for conference papers (n = 14). The 32 papers were published in a variety of journals and conferences and no preference was observed for a particular publication outlet. In specific, four journals and one conference published two or three of the selected papers each (Table 2) and all other papers were published in different journals and conferences. On the other hand, there is little variation regarding the country of the study area. The majority of studies were conducted in the US, which is probably a somewhat biased statistic, considering the large US population size, as well as the used language (e.g., English) of the study selection process. Similarly, institutions that have published more than one paper on spatial crime forecasting are based in the US with the exception of the Federal University of Rio Grande do Norte, Brazil, that has recent publications in this field.

**Table 2:** A summary of the papers’ general characteristics such as journal or conference, country of study area, institution, and scientific discipline of the first author

Top 5 journals or conferences (no of papers)	Top 3 countries (count)	
International Journal of Forecasting (3)	USA (23)	
Applied Geography (2)	Brazil (2)	
European Journal on Criminal Policy and Research (2) EPJ Data Science (2) International Conference on Systems, Man, & Cybernetics (2)	UK (2)	

Top 4 institutions (no of papers)	Top 4 disciplines (no of papers)	
University of Massachusetts Boston, US (3) Carnegie Mellon University, US (2) Federal University of Rio Grande do Norte, Brazil (2) Indiana University—Purdue University Indianapolis, US (2)	Computer Science, Information systems (12) Criminology & Penology (5)	Public administration (3) Geosciences, Multidisciplinary (2)

We also collected the discipline associated with each paper. To do so we used the affiliation of the first author and extracted the discipline down to the department level, if this was possible. Then we used as a benchmark reference the 236 categories/disciplines used in Journal Citation Reports (JCR)^1^ by the Web of Science Group. Each affiliation of authors was then matched to one of the categories. In Table 2, we see the disciplines that appeared more than one time (i.e., computer science, criminology, public administration, and geosciences). Although we collected a variety of disciplines these are the ones that we encountered more than once and account for the majority of the papers (*n *= 22). Thus scholars of these disciplines seem to have a greater interest in spatial crime forecasting.

**Fig. 2:**
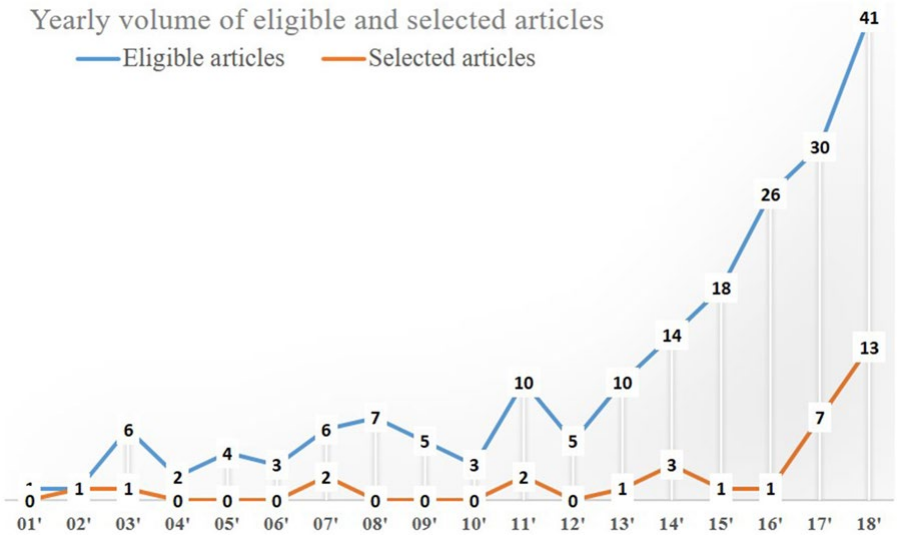
A yearly count of eligible and selected papers from 2001 to 2018

Figure 2 shows the number of eligible and selected articles by year during the study selection period. We included the eligible in addition to the selected papers for two reasons. First, many of the eligible papers looked into spatial crime forecasting but did not meet the criteria defined for this study. Second, other papers may not be relevant to forecasting, but are relevant to the broader topics of prediction or modelling. The graph in Fig. 2 depicts a rapidly increasing trend over the last couple of years. For the eligible papers, the number of articles increased substantially since 2013, whereas for the selected papers, a similar trend is evident in the last 2 years.

### Overview of selected publications on spatial crime forecasting

**Fig. 3:**
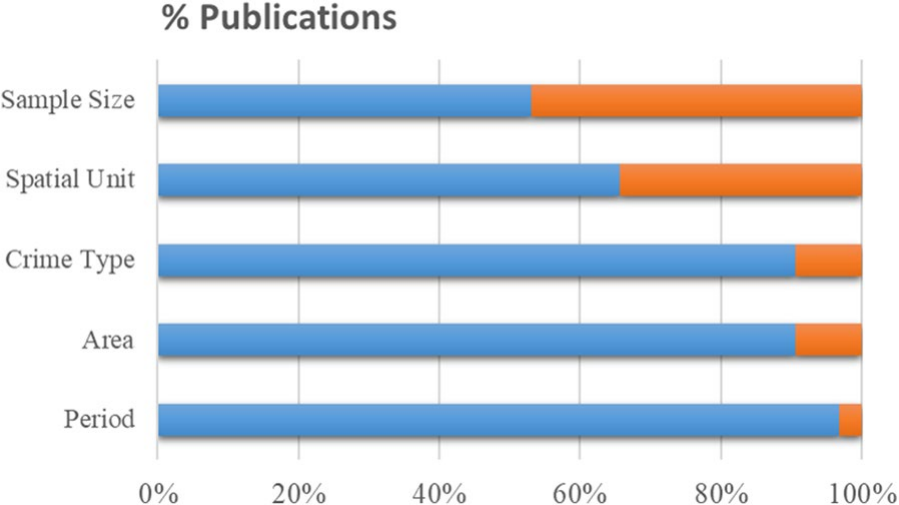
Percentages of all publications (n = 32) for describing basic information when reporting a spatial crime forecasting study. Blue: the item was properly defined; orange: the item was poorly defined or undefined

In Table 3 we enlist each selected paper along with information related to space (i.e., study area and spatial scale), time (i.e., sampling period and period in months), crime data (i.e., crime type and crime sample size), and prediction (i.e., predicted information, task, spatial unit, and temporal unit). In this section, we consider these 10 data items as initial and basic information when reporting a spatial crime forecasting study. A reader who may want to replicate or modify the methodological approach presented in the follow-up research will require the same 10 data items to assess whether such approach is adequate to the author’s follow-up study and research objectives. More importantly, when any of these data items are missing an assessment of the generalizability (or bias) of the conclusions and interpretation of results is limited. Unfortunately, the majority of the 32 selected papers (n = 21) had at least one item with undefined or unclear information for five out of the 10 data items (Fig. 3). From these, 52% (n = 11) were conference papers and 48% (n = 10) were ISI articles. On the other hand, 73% (n = 8) of the papers with no undefined or no unclear information were ISI papers and 27% (n = 3) were conference papers.

**Table 3:** An overview of the 32 selected papers with information about the space and time of the research, the crime data, and forecasting details

No*	Authors and date	Space		Time		Crime Data		Forecasting			
		Study area	Scale	Sampling period	Months	Type	Sample	Inference	Task	Spatial unit	Temporal unit
1	Araujo Junior et al. (2017)	Natal, Brazil	City	2006–2016	132	*U*	*U*	# of crimes	Regression	Rectangular grid (*U*), districts	Week
2	Araújo et al. (2018)	Natal, Brazil	City	2006–2016	132	*U*	*U*	Hotspots	Binary classification	k-means cells of varying size (*U*)	Week
3	Bowen et al. (2018)	DeKalb, USA	County	2011–2014	48	Violent crime	*U*	Hotspots	Binary classification	Census block groups	Month
4	Brown and Oxford (2001)	Richmond, USA	City	1994–1999	72	Break and enter	≈ over 24,000	# of crimes	Regression	Grid cells of 1.66 km^2^, precincts	Week, month
5	Cohen et al. (2007)	Pittsburgh, USA	City	1991–1998	96	2 crime types	1.3 million	# of crimes	Regression	1219 m × 1219 m grid cells	Month
6	Dash et al. (2018)	Chicago, USA	City	2011–2015	60	34 crime types	6.6 million	# of crimes	regression	Communities	Month, year
7	Drawve et al. (2016)	Little Rock, USA	City	2008–2009	18	Gun crime	1429	Hotspots	Binary classification	91 m × 91 m grid cells	6 months
8	Dugato et al. (2018)	Milan, Italy	City	2012–2014	36	Residential burglary	20,921	Hotspots	Binary classification	Grid cells of 2500 m^2^	Year
9	Gimenez-Santana et al. (2018)	Bogota, Colombia	city	2012–2013	24	3 crime types	*U*	Hotspots	Binary classification	75 m × 75 m grid cells	Year
10	Gorr et al. (2003)	Pittsburgh, USA	City	1991–1998	96	5 crime types	≈ 1 million	# of crimes	Regression	Police precincts	Month
11	Hart and Zandbergen (2014)	Arlington, USA	City	2007–2008	24	4 crime types	6295	Hotspots	Binary classification	Grid cells of 3 different sizes (*U*)	Year
12	Hu et al. (2018)	Baton Rouge, USA	City	2011	12	Residential burglary	3706	Hotspots	Binary classification	100 m × 100 m grid cells	Week
13	Huang et al. (2018)	New York, USA	City	2014	12	4 crime types	103,913	Category of crime	Binary classification	Districts	Day, month
14	Ivaha et al. (2007)	Cardiff, UK	City	2001–2003	26	Criminal damage	*U*	Percent of crime in clusters	Regression	Clusters of varying size (*U*)	Day
15	Johansson et al. (2015)	Sweden three cities: Stockholm, Gothenburg, and Malmö	Cities	2013–2014	12	Residential burglary	5681	Hotspots	binary Classification	Grid cells (*U*)	3 months
16	Kadar and Pletikosa (2018)	New York, USA	City	2014–2015	24	All crime and 5 crime types	174,682	# of crimes	Regression	Census tract	Year
17	Liesenfeld et al. (2017)	Pittsburgh, USA	City	2008–2013	72	All crime	9936	# of crimes	Regression	Census tracts	Month, year
18	Lin et al. (2018)	Taoyuan City, Taiwan	City	2015–2018	39	Motor vehicle thefts	≈ 8580	Hotspots	Binary classification	5 to 100 × 5 to 100 grid cells *(U)*	Month
19	Malik et al. (2014)	Tippecanoe, USA	County	2004–2014	120	all crime	≈ 310,000	Hotspots	Binary classification	Grid cells (*U*), law beats, census blocks	Week
20	Mohler (2014)	Chicago, USA	City	2007–2012	72	2 crime types	78,852	Hotspots	Binary classification	75 m × 75 m, 150 m × 150 m grid cells	Day
21	Mohler and Porter (2018)	Portland, USA	City	2012–2017	60	4 crime types	*U*	Hotspots	Binary Classification	Grid cells of 5806 m^2^	Week, 2 weeks, month, 2 months, 3 months
22	Mohler et al. (2018)	Indianapolis, USA	City	2012–2013	24	4 crime types	*U*	Hotspots	Binary classification	300 m × 300 m grid cells	Day
23	Mu et al. (2011)	Boston, USA	City	2006–2007	24	Residential burglary	*U*	Hotspots	Binary classification	20 × 20 grid cells (*U*)	Month
24	Rodríguez et al. (2017)	San Francisco, USA	City	2003–2013	120	Burglary	*U*	Properties of clusters	Regression	Clusters (*U*)	Day
25	Rosser et al. (2017)	“Major city”, UK (*U*)	City	2013–2014	13	Residential burglary	5862	Hotspots	Binary classification	Street segments (*U*)	Day
26	Rumi et al. (2018)	Brisbane, Australia; New York City, USA	Cities	2013–2013 (AUS); 2012–2013 (USA)	9 and 12	6 crime types	*U*	Hotspots	Binary classification	Census regions	3 h
27	Rummens et al. (2017)	“Large city”, Belgium (*U*)	city	2011–2014	48	3 crime types	163,800	Hotspots	Binary classification	200 m by 200 m grid cells	2 weeks, daytime month, night time month
28	Shoesmith (2013)	USA	Country	1960–2009	600	2 crime types	*U*	Crime rate	Regression	USA regions	Year
29	Yang et al. (2018)	New York, USA	city	January 2014–April 2015	16	7 crime types	*U*	Hotspots	Binary classification	0.01 latitude × 0.01 longitude grid cell size	Day, week, month
30	Yu et al. (2011)	“City in the Northeast”, USA *(U)*	City	*U*	*U*	Residential burglary	*U*	Hotspots	binary Classification	grid cells (*U*)	Month
31	Zhao and Tang (2017)	New York, USA	City	2012–2013	12	*U*	*U*	# of crimes	Regression	2 km × 2 km grid cells	Day, week
32	Zhuang et al. (2017)	Portland, USA	City	March 2012–December 2016	58	All crime	*U*	Hotspots	Binary classification	183 m × 183 m grid cells	2 weeks

*U* = undefined or unclear information

* No: Reference number of the paper that are used in Fig. 5

Most of the studies were conducted at the city level. In two studies, the forecasting area covered a county, which is the US administrative unit that usually expands across a city’s boundary. In one paper, predictions covered an entire country (US). New York City (US) was examined in four studies, Pittsburgh (US) was examined in three studies, and Portland (US), Natal (Brazil), and Chicago (US), were examined in two studies. All other publications were based on individual study areas.

The oldest crime data that were used in the 32 papers are from the year 1960 and the most recent crime data are from 2018. The sampling period ranges from 1 year up to 50 years. There is one paper with an undefined sampling period. However, from the article it can be inferred that the length of the sampling period is at least 1 year. Regarding the sample size of the crime data, it ranges from about 1400 incidents up to 6.6 million, which is relevant to the number of crime types as well as to the length of the sampling period. As for the number of crime types, there are four studies that aggregated and analysed all crime types together, twelve studies that focused on a particular crime type, fourteen studies that looked from two to up to 34 different crime types, and three studies with undefined information on the crime type analysed. Residential burglary was the crime type that was most often examined in studies that looked into only one crime type.

The last four data items in Table 3 describe details of the forecasted information, which we refer to as “inference”. The temporal unit is the time granularity of the inference and ranges from a fine granularity of 3 h up to 1 year. The most frequent temporal unit across all papers is 1 month (used in 12 papers). In addition, day and week have been used in eight studies and years in seven studies. Other less frequent temporal units are 3 h, daytime for 1 month, night-time for 1 month, 2  weeks, 2 months, 3 months, and 6 months. Similarly, the spatial unit is the space granularity of the inference and ranges from a small area of 75 m × 75 m grid cell size to a large area, such as police precincts or even countries. The spatial unit is difficult to analyse and to compare for two reasons. First, spatial units do not have a standard format like time and are two-dimensional. Thus, they can vary in size and shape. Second, for about one-third of the papers this information was poorly reported (Fig. 3). In the case of administrative units (e.g., census blocks or districts), the shape and size usually vary, but if someone is looking for further details or the units themselves, these can be in most cases retrieved by administrative authorities. However, spatial clusters may also vary in shape and size, but if details are not reported (e.g., the direction of ellipses, the range of clusters’ size, the average size of clusters) it is difficult to quantify and replicate such clusters. We also encountered cases where authors report dimensions of a grid cell size without mentioning the units of measurement. Nevertheless, the grid cell seems to be the preferable type of spatial unit and it is used in the majority of papers (n = 20).

The data items “inference” and “task” refer to the types of forecasted information and predictive task, respectively. Inference and task are defined according to the information that the authors evaluated and not to the output of a prediction algorithm. For example, an algorithm may derive crime intensity in space (i.e. the algorithm’s output), which the authors used to extract hotspots (i.e. processed output to be evaluated) from and then evaluate their results using a classification measure such as precision, accuracy, or others. Some predictive methods, such as random forest, can be used for both classification and regression tasks. It is unclear why some authors choose to apply a regression application of a method and then process, derive, and evaluate a classification output, although they could do this by directly applying a classification application of the same method. In addition, the inference “hotspots” in Table 3 includes the next four categories:

**Table:** 

1. Hotspots and non-hotspots are defined using a statistical approach that separates space between high and low crime areas.
2. Hotspots and non-hotspots are defined using an arbitrary threshold that separates space between high and low crime areas.
3. Crime and non-crime are defined using a statistical approach that separates space between areas where crime is likely to occur and areas crime is not likely to occur.
4. Crime and non-crime are defined using a statistical approach that separates space between areas where there is at least one estimated crime and areas where there is no estimated crime.

Concerning categories three and four, some authors refer to these areas as hotspots and others do not. We group all four categories together and define them as hotspots and non-hotspots, representing the output of a binary classification that separates space into areas for police patrolling that are alarming and non-alarming. We acknowledge that in the field of spatial crime analysis, hotspots are areas of high crime intensity. However, in our selected papers there does not seem to be a clear definition of the term “hotspot”.

The majority of the papers (n = 20) inferred hotspots as the outcome of a binary classification. Nine studies inferred the number of crimes or the crime rate in each spatial unit. However, three studies appear to be somehow different and unique from the rest. Huang et al. (2018) evaluated the forecasted category of crime as the output of a binary classification problem (e.g., is category A present in area X; yes or no). Ivaha et al. (2007) inferred the total number of crimes in a region, spatial clusters (or hotspots), and the share of crime within each cluster. Last, Rodríguez et al. (2017) evaluated the properties (i.e., location and size) of inferred clusters.

### Spatial crime forecasting methods

The first three data items that were extracted to be analysed in this section are the *proposed forecasting method, best proposed forecasting method*, and the *baseline forecasting method*. The latter is the method used as a comparison measure of the proposed method. We analysed the frequency of the methods for each of the three forecasting types. The *best proposed forecasting method* is the one with the best performance throughout the conducted experiments. For example, if an experiment is evaluated separately on multiple types of crimes, we only count the method with the best performance for most cases. In case two methods perform similarly (as evidenced by statistical results and reported by the authors of the papers), both methods are considered. This was necessary because some papers proposed more than one method to be compared with a baseline method, but in the end, these papers propose the method with the best performance. In addition, this reduces biased frequency counts of proposed methods. On the other hand, we considered as a baseline the method, with which the authors wanted to compare the proposed methods. For instance, Zhuang et al. (2018) proposed three Deep Neural Networks and used an additional six machine learning algorithms as baseline methods to assess how much better the proposed methods were compared to the baseline methods. In this case, we counted the six machine learning algorithms as the baseline methods.

In Table 4, we show “top” methods (i.e., frequently counted within the 32 selected papers) by each item. Random Forest (RF) is the most frequently used proposed method. Multilayer Perceptron (MLP) appears as a top method in all three items (i.e., proposed, best, baseline). Other best proposed methods are Kernel Density Estimation (KDE)-based and Risk Terrain Modelling (RTM). Interestingly, Support Vector Machines (SVM) have been proposed in five papers, but are not among the top four best-proposed methods. On the other hand, plenty well-known statistical models, are preferred as baseline methods, such as Autoregressive model (AR)-based, Logistic Regression, Autoregressive Integrated Moving Average model (ARIMA), and Linear Regression, as well as KDE-based and K Nearest Neighbours. In Additional file 1 C we added detailed tables that show for each paper the data items proposed method, best proposed method, and baseline method.

**Table 4:** Top four proposed, best proposed, and baseline methods applied in the 32 selected papers

Top 4 proposed methods (# of papers)	Top 4 best proposed methods (# of papers)	Top 4 baseline methods (# of papers)
Random Forest (7)	Random Forest (5)	Autoregressive model-based (5)
Multilayer Perceptron (6)	Multilayer Perceptron (5)	Logistic Regression (3)
Kernel Density Estimation-based (5) Support Vector Machines (5)	Kernel Density Estimation-based (5) Risk Terrain Modelling (3)	Autoregressive integrated moving average, Multilayer Perceptron, Linear Regression, KDE-based, KNN: (2)

In the next sections, we categorize the proposed forecasting methods by type of *algorithm* (“Algorithm type of proposed forecasting methods” section) and by type of *inputs* they take (“Proposed method input” section). This task was challenging because there is no scientific consensus on a single taxonomy or categorization of analytical techniques and methods. Papamitsiou and Economides (2014) reviewed papers in educational analytics, categorizing data mining methods into classification, clustering, regression, text mining, association rule mining, social network analysis, “discovery with models”, visualization, and statistics. Other researchers would summarize all of these categories, for instance, as supervised learning, non-supervised learning, and exploratory data analysis. Vlahogianni et al. (2014) use different categorizations for reviewed papers in traffic forecasting, including aspects related to the model’s approach to treating inputs and other properties relevant to split the proposed methodologies. The right granularity of properties to define a useful categorization can be problematic and particular for each field.

#### Algorithm type of proposed forecasting methods

Another suitable characteristic to classify forecasting methods is the similarities between algorithms. We divide all algorithms used in the reported papers into (i) kernel-based (ii) point process, (iii) traditional machine learning, and (iv) deep learning, according to the following criteria. Kernel-based algorithms are particularly concerned with finding a curve of crime rate $\lambda$ for each place $g$ that fits a subset of data points within the boundaries of a given kernel (see Eq. 1). We observe that the main difference among kernel-based algorithms is the use of different kernel types. Hart and Zandbergen (2014) experimented with different kernel types, providing some useful conclusions. In our selected papers, six of them have used kernel-based algorithms with the most frequently used the simple two-dimensional Kernel Density Estimation (KDE) (n = 2). However, we observed that some methods are a variation from the simple KDE model, in the form of the Spatio-Temporal KDE (STKDE) used in the paper by Hu et al. (2018), the Network-Time KDE (NTKDE) proposed by Rosser et al. (2017), or the dynamic-covariance KDE (DCKDE) proposed by Malik et al. (2014). We also have considered the Exponential Smoothing method used in the paper of Gorr et al. (2003) as a kernel-based algorithm, since it works with a window function (kernel) on time series aggregation.

1 $$\lambda_{g} \left( t \right) = \mathop \sum \limits_{{t < t_{i} }} \kappa_{g} \left( {t - t_{i} } \right)$$

Point processes can be distinguished from kernel-based algorithms insofar as a background rate factor $\mu$ that can be calculated stochastically, such as with a Poisson process, is considered. The background rate factor includes the modelling of covariates or features of the place $g$, such as demographic, economical, geographical, etc. variables (see Eq. 2). From the explanation made by Mohler (2014), we suppose that the introduction of the background rate makes the point process more suitable for multivariate modelling when compared to kernel-based algorithms. In the reviewed papers, algorithms can be distinguished among each other based on their mathematical formulations of $\kappa$ and $\mu$, but also on their internal parameter selection, mostly based on likelihood maximization. Only three papers proposed such an algorithm, including the Marked Point Process from Mohler (2014), the maximum likelihood efficient importance sampling (ML-EIS) from Liesenfeld et al. (2017), and the Hawkes Process from Mohler et al. (2018).

2 $$\lambda_{g} \left( t \right) = \mu_{g} + \mathop \sum \limits_{{t < t_{i} }} \kappa_{g} \left( {t - t_{i} } \right)$$

In the case of machine learning algorithms, their formulation is often associated with finding a function $f$ that maps feature vectors X to a given output Y. These algorithms are distinguished from each other in the way this function is estimated, some being more accurate and complex than others. We include in this category all algorithms that are explicitly associated with regression or classification. They differ from algorithms of previous categories, because $f$ is constructed only after a training process. This training step aims to find a model that minimizes the estimation error between the predicted output and the original output. The majority of the reported papers (n = 20) was included in this class of algorithms. The most proposed traditional machine learning algorithms were RF and MLP (tied at n = 6), followed by SVM together with Logistic Regression (n = 4), and Negative Binomial Regression used in RTM studies together with (n = 3).

Although deep learning algorithms have the same formulation as traditional machine learning algorithms, they present a much more complex internal structure that affects their use. The deep layer structure makes the computational budget mainly needed during training. Additionally, the need for samples is also greater, than for the other approaches. Among the reported papers, the three that have used this type of algorithm argue that it has the best overall performance. This includes the Deep Neural Networks (DNN) fitted by Lin et al. (2018), the DeepCrime framework from Huang et al. (2018), and the Long Short-Term Memory (LSTM) architecture proposed by Zhuang et al. (2017). The paper by Huang et al. (2018) even presents a neural architecture dedicated to a feature-independent approach, with a recurrent layer dedicated to encoding the temporal dependencies directly from the criminal occurrences. Still, none of these papers has discussed computational time performance against other algorithms, nor sample sizes sufficient to obtain accurate models. At the time of this writing, we argue that there is no clear guidance on when one should conduct a deep neural networks approach, although in recent years evidence of its effectiveness has begun to emerge.

#### Proposed method input

Another split factor is the inputs of the forecasting methods, i.e. the independent variables. There are some forecasting methods that accept as input the latitude, longitude, and timestamp of criminal events (raw data), while others need to apply explicit aggregations or transformations before feeding their models. In this paper, we refer as feature engineering the process of crafting, scaling and selecting covariates or features to better explain a prediction variable which often requires considerable domain-specific knowledge. An example is the aggregation of criminal events into spatiotemporal series, which can be decomposed into autoregressive lags and used as features. This feature engineering can also be applied to ancillary data methodologies not directly related to crime. For instance, Lin et al. (2018) count landmarks on the grid by counting the number of items in each cell (spatial aggregation) and craft a new feature for each landmark type, while Huang et al. (2018) define a part of their algorithm being a region embedding layer for only processing the raw location of the city’s landmarks. We believe that the split factor by method inputs may be useful information for a potential researcher who wishes to perform spatial forecasting and consults this section of our paper. Data processing requires domain knowledge, and it is an expensive (timewise) task, especially when dealing with large spatiotemporal datasets. Thus, avoiding the feature-engineering process may be preferable by some researchers. On the other hand, one may prefer to use data to derive their variables with particular patterns.

We call methods that have an internal approach to aggregating crime events into spatiotemporal variables “feature-engineering independent” and “feature-engineering dependent”. In other words, these methods explicitly need aggregations to derive spatiotemporal variables from the raw data independently of the forecasting algorithm. The majority (n = 24) of reported papers have an explicit way to transform their crime events, as well as ancillary data, into features to feed their algorithms (i.e., feature-engineering dependent). Although we have found many different forms of data aggregation into features, both spatially and temporally, the procedure of assigning features is often insufficiently reported, making it difficult to reproduce the proposed methodology. Still, well-defined workflows or frameworks followed by feature-engineering dependent methods were detailed in Malik et al. (2014) and Araújo et al. (2018). They synthesized their forecasting methods in (1) aggregate raw data spatially, following a crime mapping methodology (e.g., counting events inside grid cells), (2) generate time series and their features, (3) fit a forecasting model using an algorithm, and (4) visualize the results. In feature-engineering dependent methods the aggregation and time series generation is done separately as processing steps before fitting a model, whereas this is not needed for the feature-engineering independent methods.

### Considerations when analysing forecasting performance

In this section, we look at measures of forecasting performance (“Overview of evaluation metrics” section) and discuss which are used for each forecasting task, including for classification and regression (“Metrics by forecasting task” section). Then, we explore validation strategies by types of algorithms (“Algorithms and validation strategies” section). Finally, we summarize and discuss the main dependencies and limitations of the above subsections (“Dependencies and limitations” section).

#### Overview of evaluation metrics

As mentioned in “Spatial crime forecasting methods” section, selected papers include forecasting baseline models, novel models, or ensemble models proposed by respective authors. Evaluation metrics of such models are in general, well-known in criminology, GIScience, mathematics, or statistics. However, it is important to mention that few authors highlight the necessity of combining or using diverse evaluation metrics.

We cannot make a comparison of all evaluation results across the 32 papers due to various reasons, such as different spatial and temporal units, study areas, or forecasting methods applied. Yet, we can discuss certain similarities between them. Choosing an evaluation metric is highly dependent on the main prediction outcome, such as counts (e.g., for a Poisson distribution), normalized values or rates (e.g., for a continuous Gaussian distribution), or binary classification (crime is absent or present). The most frequent evaluation metrics used in the selected papers are the Prediction Accuracy (PA, n = 9), followed by the Prediction Accuracy Index (PAI, n = 7), the F1-Score (n = 7), Precision and Recall (n = 5), the Mean Squared Error (MSE, n = 4), the Root Mean Squared Error (RMSE, n = 3), R-squared (n = 3), the Recapture Rate Index (RRI, n = 3), the Hit Rate (n = 2), the Area Under the Curve (AUC, n = 2), and the Mean Absolute Forecast Error (MAFE, n = 2). Some additional metrics are used only once, namely the Spatio-Temporal Mean Root Square Estimate (STMRSE), the average RMSE (RMSE), the Regression Accuracy Score (RAS), the Regression Precision Score (RPS), the Ljung-Box test, the Mean Absolute Error (MAE), the Mean Absolute Percentage Error (MAPE), macro-F1, micro-F1, the Mean (Squared) Forecast Error (M(S)FE), the Pearson Correlation Coefficient, and the Nash coefficient. Generally, metrics derived from the confusion matrix, namely accuracy, precision, recall, and F1-Score, are used together to evaluate binary classifications.

We analysed the top three evaluation metrics (PA, PAI, F1-Score) in relation to their distribution among the data items of discipline, proposed forecasting algorithm type, forecasting inference, forecasting task, spatial unit, and temporal unit. Interestingly, we found that computer scientists exclusively use the PA, while criminologists prefer to apply the PAI. In addition, while the PA and the F1-Score have been preferably tested for short-term predictions (i.e., less or equal to 3 months), the PAI has been used for both short and long-term predictions. No other obvious pattern was detected among the other information elements regarding the usage and preference of these evaluation metrics.

#### Metrics by forecasting task

The most common forecasting task is binary classification (n = 21) for crime hotspots (n = 20) and the category of crime (n = 1). While the classification task is frequently discussed at the beginning of experiments, some articles consider in the performance evaluation a different item than in the output of the algorithm, thus transforming regression products into binary values. The most prominent examples include RTM models (Drawve et al. 2016; Dugato et al. 2018; Gimenez-Santana et al. 2018), where the output of the algorithm is a risk score. This score is later reclassified into a binary outcome (a positive or negative risk score) for the purpose of the evaluation. In addition, Rummens et al. (2017) propose a combined ensemble model consisting of LR and MLP that infers risk values, similar to RTM, where authors consider as crime hotspot, values with a risk higher than 20%.

The regression task (n = 11) is largely used for predicting the number of crimes (n = 8) and the performance is measured by various error measurements, such as MSE (n = 4) or RMSE (n = 3). Araujo et al. (2017) propose two new evaluation metrics, namely the Regression Accuracy Score (RAS), representing the percentage of success in predicting a sample, and the Regression Precision Score (RPS), which defines the RAS’s precision. The RPS measures the MSE of success samples normalized by the variance of the training sample (Araujo et al. (2017)). Rodríguez et al. (2017) introduce the Nash–Sutcliffe Efficiency (NSE), which they derive from hydrological models forecasting, as a normalized statistic determining the relative magnitude of the residual variance compared to the measured data variance.

However, the number of crimes is not the only inference considered in regression models. For example, Ivaha et al. (2007) predict the percentage of crime in clusters, using spatial ellipses as spatial units, Rodríguez et al. (2017) investigate properties of clusters, while Shoesmith (2013) infers crime rates from historical crime data.

**Fig. 4:**
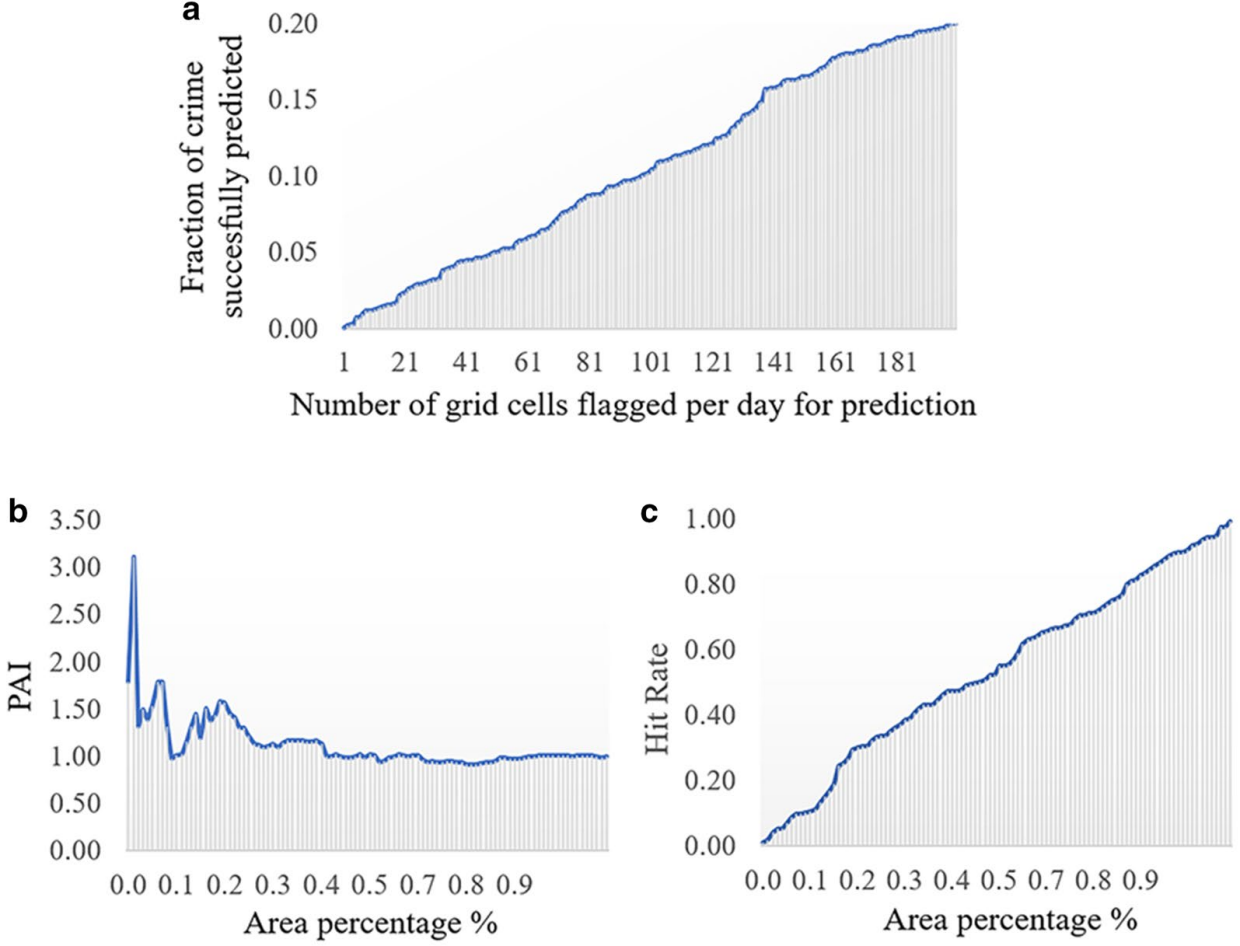
Comparable surveillance plots for evaluation metrics visualization in space (using dummy data). **a** ROC-like accuracy curve, **b** PAI curve, and **c** Hit rate curve

In addition to the above-mentioned evaluation metrics, three articles discuss surveillance plots for prediction evaluation. Mohler (2014) uses a surveillance plot metric showing the fraction of crime predicted over a time period versus the number of grid cells with real crimes for each day (Fig. 4a). The same author mentions that this metric is similar to the receiver operating characteristic curve, or ROC curve, applied by Gorr (2009), but differs because it is not using an associated false positive rate on the x-axis. Similarly, Hu et al. (2018) apply the PAI curve, also a surveillance plot showing the area percentage on the x-axis, and the PAI or the hit rate value on the y-axis (Fig. 4b, c). Similarly, Rosser et al. (2017) use hit rate surveillance plots, representing the mean hit rate against the coverage for the network and grid-based prediction approaches (Fig. 4c). These plots are highly useful to visualize metrics’ values over the surveyed territory.

#### Algorithms and validation strategies

**Fig. 5:**
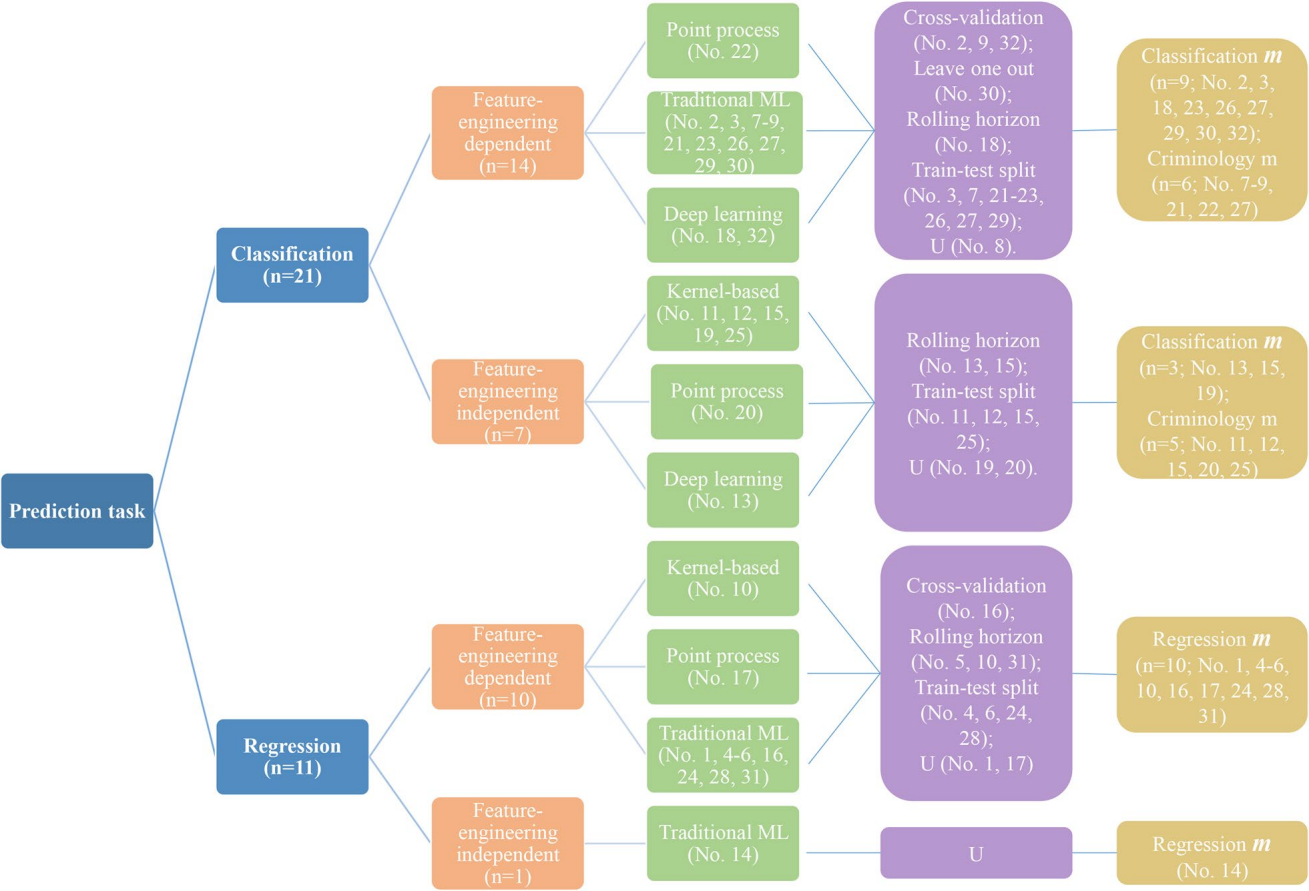
Overview of forecasting methods (see “Spatial crime forecasting methods” section) and their performance evaluation (see “Considerations when analysing forecasting performance” section) linked to the 32 selected papers. The papers’ references linked to their number are shown in Table 3. The letter ***m*** denotes an evaluation metric. The letter “U” denotes an undefined item

As mentioned in “Spatial crime forecasting methods” section, in many of the papers, the proposed forecasting method does not include a novel algorithm, but mostly applies new variables that have not previously been used before. When reminding us of the four types of algorithms, namely (i) kernel-based, (ii) point process, (iii) traditional machine learning, and (iv) deep learning, we note a diversity between the proposed forecasting and the baseline methods. In addition, validation strategies are diverse, as well. Half of the studies (n = 16) consider splitting the data into training and testing subsets. Most of these studies include 70% training (current) for 30% testing (future) sets. Johansson et al. (2015) use a combined approach, including rolling horizon, which is producing ten times the size of a sample for the KDE algorithm, containing 70% of the original crime dataset (keeping the 70/30 ratio). The final result is calculated as the mean of the ten measurements. Figure 5 gives a good overview of all algorithms and their validation strategies. This decision tree visualization includes five central data items, namely prediction task, proposed input forecasting method, proposed forecasting algorithm type, validation strategy, and evaluation metrics. Classification ***m*** refers to those evaluation metrics that are particularly used for classification tasks (e.g., PA, F1-score). Regression ***m*** is a composition of error metrics for regression analysis (e.g., MSE, RMSE, MAE), while Criminology ***m*** includes crime analysis metrics (e.g., PAI, RRI).

Kernel-based algorithms are preferably used to predicting hotspots (n = 5) and the number of crimes (n = 1). Interestingly, Malik et al. (2014) bring into discussion the fact that regions with similar demographics tend to illustrate similar trends for certain crime types. This observation is included in their prediction model “Dynamic Covariance Kernel Density Estimation method (DSKDE)” and compared with the “Seasonal Trend decomposition based on Loess (STL)” baseline model. Hart and Zandbergen (2014) and Johansson et al. (2015) use a kernel-based KDE approach without comparing it with a baseline method, both considering the PAI as one of the evaluation metrics. Only two of the kernel-based studies consider ancillary data (Gorr et al. 2003; Rosser et al. 2017), yet both use different validation strategies (rolling-horizon and train-test split, respectively) and evaluation metrics (MAE, MSE, MAPE in the first publication and Hit Rate in the second publication). Thus, it is worth noting that, while using the same base algorithm, such as KDE, other components of the prediction process may be different.

Two out of three point process algorithms do not explain the validation strategy followed in the studies (Liesenfeld et al. 2017; Mohler 2014). Mohler (2014) shows an interesting point process approach using only historical crime data, capturing both short-term and long-term patterns of crime risk. This article includes the surveillance plot evaluation (see “Metrics by forecasting task” subsection), comparing chronic and dynamic hotspot components for homicides and all crime types.

The third category of forecasting algorithms, the traditional ML, is split up almost equally between classification and regression tasks. Only three articles discussing traditional ML algorithms do not mention information about the baseline comparison (Araújo et al. 2018; Rodríguez et al. 2017; Rummens et al. 2017). The majority of ML algorithms (n = 11) use the training–testing split validation strategy applied to the classification task. Interestingly, one of the articles (Yu et al. 2011) discusses a different validation approach, the “Leave-One-Month-Out” (LOMO), where instead of running the classification only once on the training and testing data sets, it is run on S − 1 sets (S = number of sets/months).

An increasing body of forecasting techniques are based on DL, however, for this review, we include only three articles, with all of them for short-term prediction and coming from the computer science discipline (Huang et al. 2018; Lin, Yen, and Yu 2018; Zhuang et al. 2017). Two of the three articles consider geographic ancillary variables and apply the rolling-horizon validation strategy, while the third article deals only with crime lags following a 10-fold cross-validation approach. All three articles consider a binary classification evaluated by metrics such as the PA and the F1-score. Zhuang et al. (2017) propose a spatio-temporal neural network (STNN) for forecasting crime occurrences, while embedding spatial information. They then compare the STNN with three state-of-the-art methods, including the Recurrent Neural Network (RNN), the Long Short-Term Memory (LSTM), and the Gated Recurrent Unit (GRU). Since the model is designed for all types of crime data, each crime type can lead to different performances of the STNN due to their variability in time and space. Presumably, challenges will appear for crime types with low data volumes, because neural networks require a sufficient amount of data for training.

#### Dependencies and limitations

Although most papers use standard evaluation metrics, such as PA for a binary outcome, they usually do not include complementary metrics, in order to ensure that every aspect of the prediction performance is covered. Often, the PA is used by itself to measure model performance (Araújo et al. 2018; Malik et al. 2014; Mu et al. 2011). Complementary metrics are needed, because whilst one method may have a higher evaluation score than others, they may provide additional information. For example, while showing a high PAI, the Prediction Efficiency Index (PEI) value (Hunt 2016) may be reduced. PEI is another evaluation metric that is calculated by the ratio of the PAI to the maximum possible PAI a model can achieve. The difference between the PAI and the PEI can be explained by both metrics having different dependencies on the cell size.

Complementary metrics also overcome limitations of some evaluation measures. For example, the PA is the sum of true positives and true negatives divided by the total number of instances, which represents the percentage that is classified correctly. However, this information may not be enough to judge the performance of a model, because it omits information about precision. The Hit rate and the PAI are obtained through a division, thus, when the denominator is small, both metrics are high. Consequently, when crime occurrences are low, results are heavily affected.

Furthermore, traditional metrics are global in nature, but in spatial prediction or forecasting, we are also interested in the spatial distribution of the prediction. There may be local areas of good and bad prediction performance, resulting in an average global value. A complementary metric for a regression outcome could be to calculate the Moran’s I of the prediction error and explore the variation of the predictive performance throughout the study area. Ideally, the prediction error should follow a random spatial distribution. Overall, we find a low to no interest in developing (local) spatial, temporal, or spatiotemporal evaluation metrics.

The relevance of evaluation metrics may be biased for various reasons. One example can be the class imbalance. A model can have high accuracy while predicting the locations without crime very well. In contrast, locations with crimes are not well forecasted. Some authors try to ameliorate the negative–positive ratio between crime and no crime cells, by adjusting the weight of hotspots and cold spots (Yu et al. 2011), or change the training set, while the test set keeps its original, real data (Rumi et al. 2018). Another dependency is the different kinds of aggregation that take place during modelling by time, space, or crime types attributes. For instance, while the majority of papers report to work with disaggregated crime types, some of them consider to aggregate crime types to, e.g., “violent crimes”, without specifying which types are included. In addition, the effects spatiotemporal aggregations have on the forecasting performance are typically not analysed, but could easily be conducted with a sensitivity analysis.

## Discussion

In this section, we perform a SWOT analysis of the most significant findings.

### Strengths

One of the strongest elements of current research efforts is the incorporation of spatial or spatiotemporal information into traditional prediction algorithms. The examples of this approach is STAR and STKDE (Shoesmith 2013; Rosser et al. 2017). Also, KDE, a traditional method in the field, has been adapted to consider sampling problems, such as sparse data (DCKDE) and grid format (NTKDE) (Malik et al. 2014; Rosser et al. 2017). Besides, the interest of the scientific community in the incorporation and effect of big data into prediction is evident from the related work section. This interest is also supported by the trend of introducing dynamic variables into the modelling process, such as calculating visitor entropy from Foursquare or ambient population from social networks and transportation. Regarding the performance evaluation, surveillance plots (Fig. 4) provide a more detailed picture of the accuracy of the forecasted information. Since they include the area coverage on the x-axis, they can be used by the police as a decision tool to identify the threshold that balances prediction accuracy with the size of patrolling areas.

### Weaknesses

Overall, significant details of study experiments are not always reported and commonly undefined items are the spatial unit of analysis and the sample size. Similarly, for feature-engineering dependent methods the crafting procedures are not sufficiently described. The above elements make a study difficult to reproduce or to compare its results with a possible future study. Furthermore, we did not find any open source tools that implement spatial crime forecasting using the best-proposed methods reported. Such a tool could enhance the possibility of reproducing results from an existing forecasting study. We suggest that all data items analysed in “Overview of selected publications on spatial crime forecasting” section (for an overview have a look at Table 3) should always be reported. However, a detailed “spatial forecasting protocol” could be developed similarly to protocols for other modelling approaches such as the ODD protocol (Grimm et al. 2010). Furthermore, the most common spatial unit is the grid cell, which may not necessarily align with places that policing resources are typically deployed to. So far, we did not encounter a study that sufficiently addresses this issue. Regarding the performance evaluation, most authors use standard metrics. A “global” standard metric, such as MAE, cannot describe the distribution of the prediction error across space, which can vary a lot. We thus propose to develop novel local spatial or spatiotemporal evaluation metrics. Finally, other modelling issues are hardly discussed, if at all, such as overfitting, multi-collinearity, sampling bias, and data sparsity.

### Opportunities

There is a tremendous increase in spatial crime forecasting studies. From the pool of the 32 selected papers, 7 and 11 papers were published in 2017 and 2018, respectively, compared to about one paper per year between 2000 and 2016 (Fig. 2). This shows the growing interest of scholars from varying disciplines (compare Table 2) into this kind of research. The crime type that has been studied the most is residential burglary. It is unclear why this particular crime type and property crimes, in general, are more likely to be studied. A future opportunity could be to systematically test whether there is a pattern of property crimes to consistently outperforming other crime types and why. Furthermore, except for RTM and KDE, other spatial methods mentioned in the related work section (“Related work” section) were not used by the selected papers. The reason may be that authors have varying backgrounds, such as computer science and criminology, and may not be familiar with such methods. This opens a research opportunity to explore and compare less used spatial methods with traditional approaches, such as RTM or KDE. Another opportunity would be to compare the forecasting performance of the methods among each other. In this review, we presented methodological trends, but a fair comparison among spatial methods was not possible. First, some methods were not compared to a baseline method. Other authors compared the same method with a different set of features. Even if there were papers with a similar set of features a comparison among them would be biased due to variations of sample data, study areas, sampling periods, etc. Future empirical studies should focus on the comparison of algorithms, of which the number is constantly increasing. We merged the selected papers into four categories of forecasting algorithms, including the kernel-based, point processes, traditional machine learning, and deep learning. Traditional machine learning algorithms were present in most proposed methods, with MLP and RF being the most common ones, while AR models were the most used baselines methods. A suggestion is to compare new or recently developed algorithms to the most frequently proposed ones, instead of continuing to conduct further comparisons with traditional or simpler methods that have repeatedly shown to underperform.

### Threats

We outlined that spatial crime forecasting studies lack coherent terminology, especially for terms such as “prediction”, “forecasting”, and “hotspots”. The predominant predictive task is the binary classification (n = 21) and the predominant forecasting inference is hotspots (n = 20). It is important to understand the rationale behind this trend. Is regression analysis less useful or more difficult to predict? Although we notice a constant increase in developing classification algorithms or features to be infused in the classification task, we acknowledge the importance of both prediction tasks. Also, for the display of an area’s crime picture, it is important to examine both hotspots and coldspots or a multiclass classification towards the hottest crime spots. However, none of these was the focus of the examined papers. We acknowledge that forecasting hotspots is important for police to allocate resources. Nevertheless, what about the information that can be derived by other types of spatial groupings such as coldspots, coldspot outliers, or hotspot outliers, commonly referred to as LL, LH, HL (low–low, low–high, high-low, respectively) and calculated by the local Moran statistic (Anselin 2005)? Science needs to progress knowledge, which requires understanding and examining all aspects of a phenomenon. Finally, only a third of all papers performed long-term predictions. Although this trend is positive because law enforcement has an interest in almost real-time prediction, the long-term prediction should not be overlooked as playing an important role in the understanding of the crime risk and providing a broad picture for strategic planning.

## Conclusion

In this paper, we focus on “Spatial Crime Forecasting”, which is an inference approach about crime both in time and in space. We conducted a systematic literature review that follows the reporting guidance “PRISMA” (Liberati et al. 2009) to understand and evaluate the state of the art concerning concepts and methods in empirical studies on crime with many applications and special attention to crime. We addressed several research questions that deal with the role of space in the forecasting procedure, the methods used, the predictive performance, and finally model validation strategies.

We identified five types of inference, namely (1) hotspots (the majority of the papers), (2) number of crime, (3) crime rate, (4) category of crime, (5) percent of crime in clusters, and (6), properties of clusters. With regards to forecasting methods, the authors proposed mostly traditional machine learning methods, but also kernel density estimation based approaches, and less frequently point process and deep learning approaches. When it comes to measuring performance, a plethora of metrics were used with the top three ones being the Prediction Accuracy, followed by the Prediction Accuracy Index, and the F1-Score. Finally, the most common validation approach was the train-test split while other approaches include the cross-validation, the leave one out, and the rolling horizon.

This study was driven by the increasing publication of spatial crime forecasting studies and (crime predictive analytics in general). More than half of the selected papers (n = 32) were published in the last 2 years. In specific, about one paper per year was published between 2000 and 2016, while 7 and 11 papers were published in 2017 and 2018, respectively. At the same time, there is a global growth of scientific publication outputs. Bornmann and Mutz (2015), fitted an exponential model to this growth and calculated an increasing rate of outputs of about 3% annually, while the volume is estimated to double in approximately 24 years. Yet the yearly patterns of the selected papers show a much greater increase that indicates the importance and future potential of studies related to spatial crime forecasting.

Furthermore, we would like to outline the main limitations that may prohibit reproducibility, and hence the advancement of this topic in the long term. First, the terminology being used is not consistent possibly due to the fact that scientists working on this topic have various backgrounds (e.g. criminology, computer science, geosciences, public policy, etc.). Second, significant details of study experiments are vaguely or not at all reported. With respect to the last point, we suggested reporting the following data items: *study area, scale, sampling period, months, type, sample, inference, task, spatial unit, and temporal unit* (in total 10 items). Additional items to be reported are *proposed method, best*-*proposed method, baseline method, evaluation metrics, and validation strategy* (in total 5 items).

## Supplementary information

**Additional file 1.** Online survey on Risk of Bias across Studies.

## Data Availability

The list of manuscripts used for this research is mentioned in Table 3. If needed, the authors can provide the list of 193 manuscripts that went through the eligibility phase.

## Footnotes

1 JCR: https://clarivate.com/webofsciencegroup/solutions/journal-citation-reports/.

## References

[CR7] Al Boni, M., & Gerber, M. S. (2016). Predicting crime with routine activity patterns inferred from social media. In *IEEE International Conference on Systems, Man and Cybernetics (SMC)*, (pp. 1233–1238). https://ieeexplore.ieee.org/stamp/stamp.jsp?arnumber=7844410.

[CR2] Anselin L (2005). Exploring spatial data with GeoDaTM: A workbook.

[CR3] Araújo, A., Cacho, N., Bezerra, L., Vieira, C., & Borges, J. (2018). Towards a crime hotspot detection framework for patrol planning. In *2018 IEEE 20th International Conference on High Performance Computing and Communications; IEEE 16th International Conference on Smart City; IEEE 4th International Conference on Data Science and Systems (HPCC/SmartCity/DSS)*, (pp. 1256–1263). 10.1109/HPCC/SmartCity/DSS.2018.00211.

[CR4] Araujo, A. J., Cacho, N., Thome, A. C., Medeiros, A., & Borges, J. (2017). A predictive policing application to support patrol planning in smart cities. In *International Smart Cities Conference (ISC2)*. https://www.researchgate.net/profile/Adelson_Araujo2/publication/321236214_A_predictive_policing_application_to_support_patrol_planning_in_smart_cities/links/5c068339299bf169ae316a6f/A-predictive-policing-application-to-support-patrol-planning-in-smart-ci.

[CR6] Bernasco W, Elffers H, Piquero AR, Weisburd D (2010). Statistical analysis of spatial crime data. Handbook of quantitative criminology.

[CR8] Bornmann L, Mutz R (2015). Growth Rates of Modern Science: A Bibliometric Analysis Based on the Number of Publications and Cited References. Journal of the Association for Information Science and Technology.

[CR200] Bowen DA, Mercer Kollar LM, Wu DT, Fraser DA, Flood CE, Moore JC, Mays EW, Sumner SA (2018). Ability of crime, demographic and business data to forecast areas of increased violence. International journal of injury control and safety promotion.

[CR9] Bramer WM, Rethlefsen ML, Kleijnen J, Franco OH (2017). Optimal database combinations for literature searches in systematic reviews: A prospective exploratory study. Systematic Reviews.

[CR10] Brantingham PJ, Brantingham PL (1984). Patterns in crime.

[CR11] Brayne S (2017). Big data surveillance: The case of policing. American Sociological Review.

[CR201] Brown, D. E., & Oxford, R. B. (2001). Data mining time series with applications to crime analysis. In *2001 IEEE International Conference on Systems, Man and Cybernetics. e-Systems and e-Man for Cybernetics in Cyberspace* (Cat. No. 01CH37236), Vol. 3 (pp. 1453–1458). IEEE. 10.1109/ICSMC.2001.973487.

[CR12] Bruinsma GJN, Johnson SD (2018). The oxford handbook of environmental criminology.

[CR13] Caplan JM, Kennedy LW, Miller J (2011). Risk terrain modeling: brokering criminological theory and gis methods for crime forecasting. Justice Quarterly.

[CR14] Chainey S, Tompson L, Uhlig S (2008). The utility of hotspot mapping for predicting spatial patterns of crime. Security Journal..

[CR15] Chauhan, C., & Sehgal, S. 2017. A review: crime analysis using data mining techniques and algorithms. In P. N. Astya, A. Swaroop, V. Sharma, M. Singh, & K Gupta, (Ed.), *2017 IEEE International Conference on Computing, Communication and Automation (ICCCA)*, edited by , (pp. 21–25).

[CR16] Chen HC, Chung WY, Xu JJ, Wang G, Qin Y, Chau M (2004). Crime data mining: A general framework and some examples. Computer.

[CR202] Cohen J, Gorr WL, Olligschlaeger AM (2007). Leading indicators and spatial interactions: A crime‐forecasting model for proactive police deployment. Geographical Analysis.

[CR17] Cressie NAC (1993). Statistics for spatial data.

[CR203] Dash, S. K., Safro, I., & Srinivasamurthy, R. S. (2018). Spatio-temporal prediction of crimes using network analytic approach. In *2018 IEEE International Conference on Big Data (Big Data)* (pp. 1912-1917). IEEE. 10.1109/BigData.2018.8622041.

[CR18] Drawve G, Moak SC, Berthelot ER (2016). Predictability of gun crimes: A comparison of hot spot and risk terrain modelling techniques. Policing & Society.

[CR19] Dugato M, Favarin S, Bosisio A (2018). Isolating target and neighbourhood vulnerabilities in crime forecasting. European of Criminal Policy and Reserach..

[CR20] Gerber MS (2014). Predicting crime using twitter and kernel density estimation. Decision Support Systems..

[CR21] Gimenez-Santana A, Caplan JM, Drawve G (2018). Risk terrain modeling and socio-economic stratification: Identifying risky places for violent crime victimization in Bogota, Colombia. European of Criminal Policy and Reserach.

[CR22] Gorr WL (2009). Forecast accuracy measures for exception reporting using receiver operating characteristic curves. International Journal of Forecasting.

[CR23] Gorr, W., & Harries, R. (2003). Introduction to crime forecasting. *International Journal of Forcasting* 19. https://www.sciencedirect.com/science/article/pii/S016920700300089X.

[CR24] Gorr, W., Olligschlaeger, A., & Thompson, Y. International Journal Of, and Undefined 2003. (2003). “Short-Term Forecasting of Crime.” *International Journal of Forecasting*. https://www.sciencedirect.com/science/article/pii/S016920700300092X.

[CR25] Grimm V, Berger U, DeAngelis DL, Polhill JG, Giske J, Railsback SF (2010). The ODD protocol: A review and first update. Ecological Modelling.

[CR26] Haddaway, N. R., Collins, A. M., Coughlin, D., & Kirk, S. (2015). The role of google scholar in evidence reviews and its applicability to grey literature searching. *PloS ONE* 10(9). 10.1371/journal.pone.0138237 PMC4574933 26379270

[CR27] Hardyns W, Rummens A (2018). predictive policing as a new tool for law enforcement? Recent developments and challenges. European Journal on Criminal Policy and Research.

[CR28] Hart T, Zandbergen P (2014). Kernel density estimation and hotspot mapping examining the influence of interpolation method, grid cell size, and bandwidth on crime forecasting. Policing—An International Journal o FPolice Strategies & Management.

[CR29] Hassani H, Huang X, Silva ES, Ghodsi M (2016). A review of data mining applications in crime. Statistical Analysis and Data Mining.

[CR30] Holone H (2016). The filter bubble and its effect on online personal health information. Croatian Medical Journal.

[CR31] Hu Yujie, Wang Fahui, Guin Cecile, Zhu Haojie (2018). A spatio-temporal kernel density estimation framework for predictive crime hotspot mapping and evaluation. Applied Geography.

[CR32] Huang, C., Zhang, J., Zheng, Y., & Chawla, N. V. (2018). DeepCrime: Attentive hierarchical recurrent networks for crime prediction. In *Proceedings of the 27th ACM International Conference on Information and Knowledge Management*, (pp. 1423–1432). CIKM’18. New York, NY, USA: ACM. 10.1145/3269206.3271793.

[CR33] Hunt, J. M. (2016). Do crime hot spots move? Exploring the effects of the modifiable areal unit problem and modifiable temporal unit problem on crime hot spot stability. American University.

[CR34] Ivaha, C., Al-Madfai, H., Higgs, G., & Ware, J. A. (2007). The dynamic spatial disaggregation approach: A spatio-temporal modelling of crime. In *World Congress on Engineering*, (pp. 961–966). Lecture Notes in Engineering and Computer Science. http://www.iaeng.org/publication/WCE2007/WCE2007_pp961-966.pdf.

[CR35] Johansson, E., Gåhlin, C., & Borg, A. (2015). Crime hotspots: An evaluation of the KDE spatial mapping technique. In *2015 European Intelligence and Security Informatics Conference*, (pp. 69–74). 10.1109/EISIC.2015.22.

[CR36] Kadar, C., Brüngger, R. R., & Pletikosa, I. (2017). Measuring ambient population from location-based social networks to describe urban crime. In *International Conference on Social Informatics*, (pp. 521–35). Springer, New York.

[CR37] Kadar C, Pletikosa I (2018). Mining large-scale human mobility data for long-term crime prediction. EPJ Data Science.

[CR38] Kennedy LW, Caplan JM (2012). A theory of risky places.

[CR39] Kennedy LW, Dugato M (2018). “Forecasting crime and understanding its causes. Applying risk terrain modeling worldwide. European Journal on Criminal Policy and Research.

[CR40] Kinney JB, Brantingham PL, Wuschke K, Kirk MG, Brantingham PJ (2008). Crime attractors, generators and detractors: Land use and urban crime opportunities. Built Environment.

[CR41] Liberati A, Altman D, Tetzlaff J, Mulrow C, Gøtzsche PC, Ioannidis JPA, Clarke M, Devereaux PJ, Kleijnen J, Moher D (2009). The PRISMA statement for reporting systematic reviews and meta-analyses of studies that evaluate health care interventions: Explanation and elaboration. Journal of Clinical Epidemiology.

[CR42] Liesenfeld R, Richard JF, Vogler J (2017). Likelihood-based inference and prediction in spatio-temporal panel count models for urban crimes. Journal of Applied Econometrics.

[CR43] Lin YL, Yen MF, Yu LC (2018). Grid-based crime prediction using geographical features. ISPRS International Journal of Geo-Information.

[CR44] Malik, A., Maciejewski, R., Towers, S., McCullough, S., & Ebert, D. S. (2014). Proactive spatiotemporal resource allocation and predictive visual analytics for community policing and law enforcement. *IEEE Transactions on Visualization and Computer* 20(12): 1863–72. https://www.computer.org/csdl/trans/tg/2014/12/06875970-abs.html. 10.1109/TVCG.2014.2346926 26356900

[CR45] Mohler G (2014). Marked point process hotspot maps for homicide and gun crime prediction in Chicago. International Journal of Forecasting.

[CR204] Mohler G, Porter MD (2018). Rotational grid, PAI-maximizing crime forecasts. Statistical Analysis and Data Mining: The ASA Data Science Journal.

[CR46] Mohler, G., Raje, R., Carter, J., Valasik, M., & Brantingham, J. (2018). A penalized likelihood method for balancing accuracy and fairness in predictive policing. In *2018 IEEE International Conference on Systems, Man, and Cybernetics (SMC)*. https://ieeexplore.ieee.org/abstract/document/8616417/.

[CR47] Mohler GO, Short MB, Brantingham PJ, Schoenberg FP, Tita GE (2011). Self-exciting point process modeling of crime. Journal of the American Statistical Association.

[CR48] Mohler GO, Short MB, Malinowski S, Johnson M, Tita GE, Bertozzi AL, Brantingham PJ (2015). Randomized controlled field trials of predictive policing. Journal of the American Statistical Association.

[CR205] Mu Yang, Ding Wei, Morabito Melissa, Tao Dacheng (2011). Empirical Discriminative Tensor Analysis for Crime Forecasting. Knowledge Science, Engineering and Management.

[CR49] Ohyama T, Amemiya M (2018). applying crime prediction techniques to Japan: A comparison between risk terrain modeling and other methods. European Journal on Criminal Policy and Research.

[CR50] Ozkan T (2018). Criminology in the age of data explosion: new directions. The Social Science Journal.

[CR51] Papamitsiou Z, Economides AA (2014). Learning analytics and educational data mining in practice: A systematic literature review of empirical evidence. Journal of Educational Technology & Society.

[CR52] Perry WL (2013). Predictive policing: The role of crime forecasting in law enforcement operations.

[CR53] Ratcliffe J (2015). What is the future… of predictive policing. Practice.

[CR54] Rodríguez, C. D., Gomez, D. M., & Rey, M. A. (2017). Forecasting time series from clustering by a memetic differential fuzzy approach: An application to crime prediction. In *2017 IEEE Symposium Series on Computational Intelligence (SSCI)*, (pp. 3372–3379). https://ieeexplore.ieee.org/abstract/document/8285373.

[CR55] Rosser G, Davies T, Bowers KJ, Johnson DS, Cheng T (2017). Predictive crime mapping: Arbitrary grids or street networks?. Journal of Quantitative Criminology.

[CR56] Rumi, S. K., Deng, K., & Salim, F. D. EPJ Data Science, and Undefined 2018. (2018). Crime event prediction with dynamic features. *EPJ Data Science*. 10.1140/epjds/s13688-018-0171-7.

[CR57] Rummens A, Hardyns W, Pauwels L (2017). The use of predictive analysis in spatiotemporal crime forecasting: Building and testing a model in an urban context. Applied Geography.

[CR58] Seele P (2017). Predictive sustainability control: A review assessing the potential to transfer big data driven ‘predictive policing’ to corporate sustainability management. Journal of Cleaner Production.

[CR59] Shamsuddin, N.H. M., Ali. N. A., & Alwee, R. (2017). An overview on crime prediction methods. In *6th ICT International Student Project Conference (ICT*-*ISPC), IEEE.* https://ieeexplore.ieee.org/abstract/document/8075335/.

[CR60] Shoesmith GL (2013). Space–time autoregressive models and forecasting national, regional and state crime rates. International Journal of Forecasting.

[CR61] Thongsatapornwatana, U. (2016). A survey of data mining techniques for analyzing crime patterns. In *2016 Second Asian Conference on Defence Technology (ACDT)*, (pp. 123–28). https://ieeexplore.ieee.org/stamp/stamp.jsp?tp=&arnumber=7437655.

[CR62] Thongtae, P., & Srisuk, S. (2008). An analysis of data mining applications in crime domain. In X. He, Q. Wu, Q. V. Nguyen, & W. Ja (Ed.), *8th IEEE International Conference on Computer and Information Technology Workshops*, (pp. 122–126). 10.1109/CIT.2008.Workshops.80.

[CR63] Vlahogianni EI, Karlaftis MG, Golias JC (2014). Short-term traffic forecasting: where we are and where we’re going. Transportation Research Part C: Emerging Technologies.

[CR64] Wang, X., & Brown, D. E. (2011). The Spatio-Temporal Generalized Additive Model for Criminal Incidents. In *Proceedings of 2011 IEEE International Conference on Intelligence and Security Informatics*, (pp. 42–47). IEEE, New York.

[CR65] Wang, X., Brown, D. E., Gerber, M.S. (2012). Spatio-temporal modeling of criminal incidents using geographic, demographic, and twitter-derived information. In *2012 IEEE International Conference on Intelligence and Security Informatics*, (pp. 36–41). IEEE, New York.

[CR66] Wang, H., Kifer, D., Graif, C., & Li, Z. (2016). Crime rate inference with big data. In *Proceedings of the 22nd ACM SIGKDD International Conference on Knowledge Discovery and Data Mining*, (pp. 635–644). KDD’16. New York, NY, USA: ACM. 10.1145/2939672.2939736.

[CR67] Williams ML, Burnap P (2015). Cyberhate on social media in the aftermath of woolwich: A case study in computational criminology and big Data. British Journal of Criminology.

[CR206] Yang D, Heaney T, Tonon A, Wang L, Cudré-Mauroux P (2018). CrimeTelescope: crime hotspot prediction based on urban and social media data fusion. World Wide Web.

[CR68] Yu, C. H., Ward, M. W., Morabito, M., & Ding, W. (2011). Crime forecasting using data mining techniques. In *IEEE 11th International Conference on Data Mining Workshops*, (pp. 779–786). IEEE, New York.

[CR69] Zhao, X., & Tang, J. (2017). Modeling temporal-spatial correlations for crime prediction. In *Proceedings of the 2017 ACM on Conference on Information and Knowledge Management*, (pp. 497–506). CIKM’17. New York, NY, USA: ACM. 10.1145/3132847.3133024.

[CR70] Zhuang, Y., Almeida, M., Morabito, M., & Ding. W. (2017). Crime hot spot forecasting: A recurrent model with spatial and temporal information. In X. D, Wu, T. Ozsu, J. Hendler, R. Lu, (Ed.), *IEEE International Conference on Big Knowledge (ICBK)*, (pp. 143–150). 10.1109/ICBK.2017.3.

