# Supplementary material for: A systematic review on spatial crime forecasting
Source: Crime Sci. 2020 May 27;9(1):7. doi: 10.1186/s40163-020-00116-7 (PMC7319308; doi:10.1186/s40163-020-00116-7)
Supplement: Supplementary file 1 — Additional file 1. Online survey on Risk of Bias across Studies. [file 40163_2020_116_MOESM1_ESM.docx]

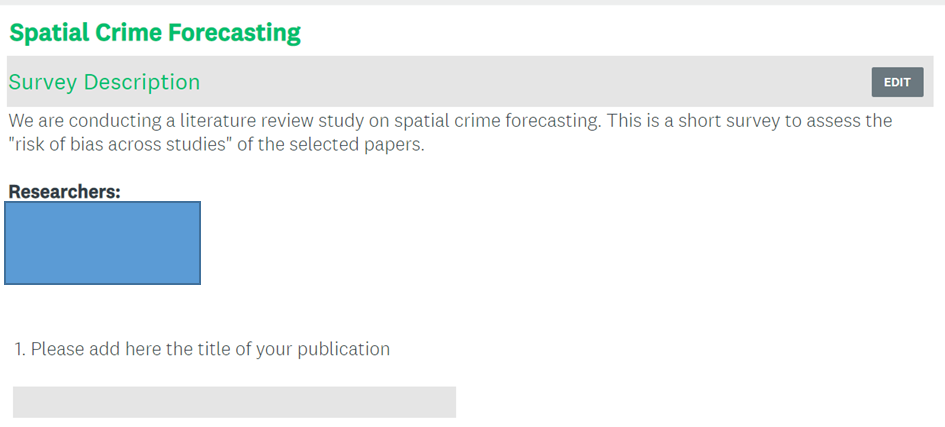

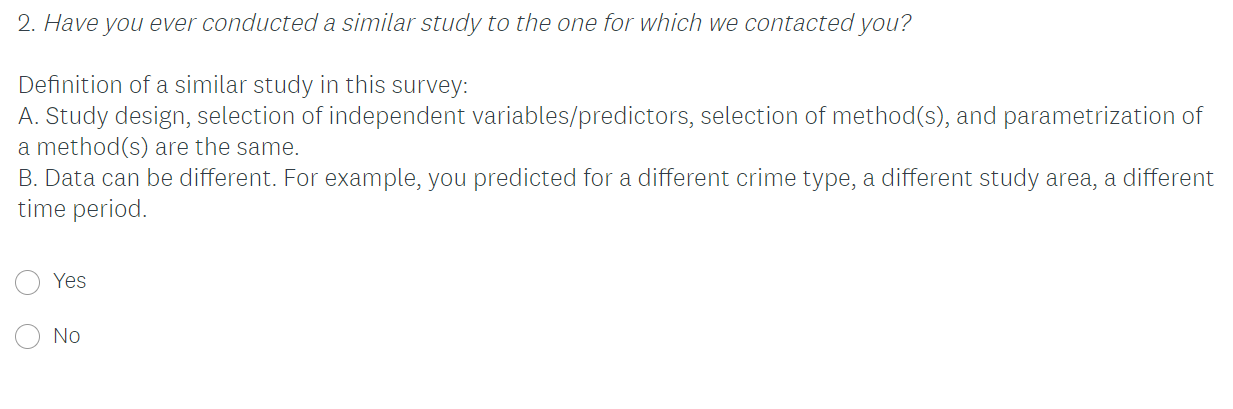

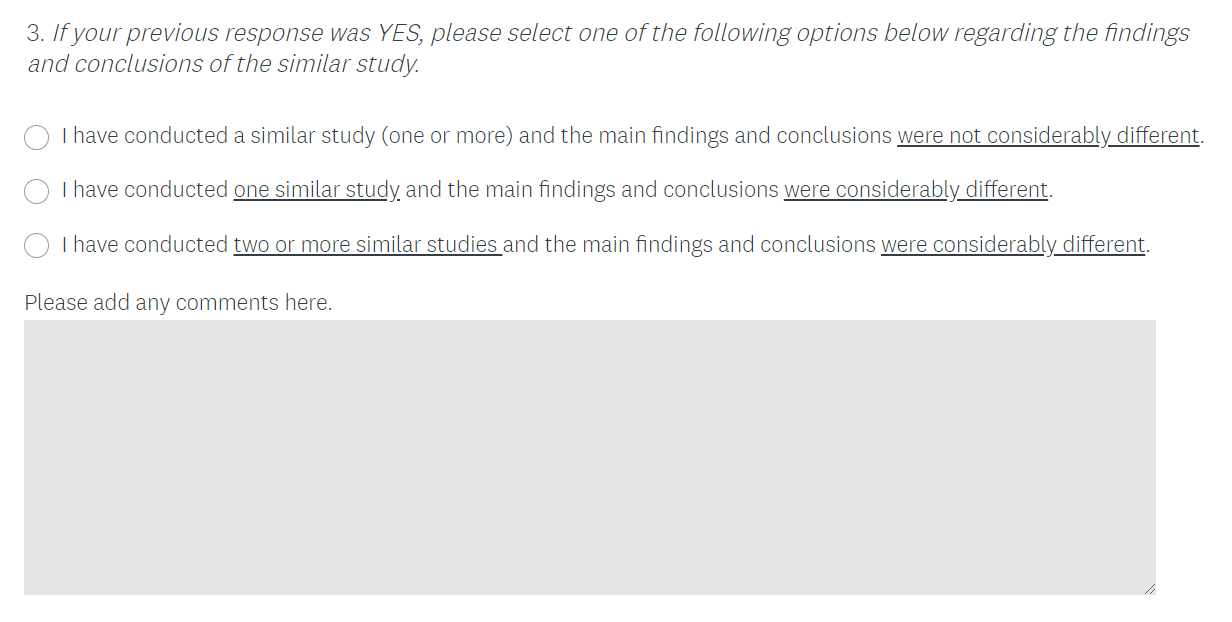


## Additional file A: Online survey on Risk of Bias across Studies

*Total responses: 13, Valid: 12*

*Number of papers addressed in the survey:* 11

*Typical time spent 2m: 18s*

*Period of responses: 16/5/2019 – 17/6/2019*

## Additional file B: A word cloud of the titles of the selected studies


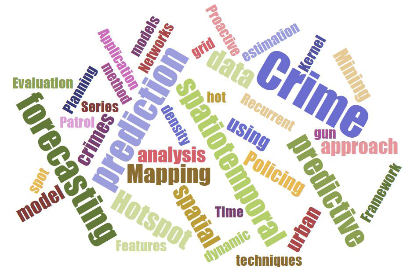
Figure 1 shows a cloud of the forty words that appear most often in the titles of these papers. A different color is given to each word, but the size of the font increases with the number of times that this word appears in the titles. Crime, forecasting, prediction, predictive, spatial, temporal, spatiotemporal, hotspot, and data were words that appeared five or more times. These words were used as keywords in the Identification phase. Most of the keywords that we used (18 in total) are depicted in this word cloud with the exception of four phrases. These are big data, machine learning, risk terrain modelling, and near-repeat crime. For the first three keywords, we show in the results sections of the manuscript that they are significant components of the research content of the selected papers, although they do not appear in the titles. As for the last phrase, near-repeat crime, we seem to have overestimated its contribution to the finally selected papers.

Fig. S1: A word cloud of high frequency words (i.e., the top 40) extracted from the titles of the selected papers.

## Additional file C: Proposed, Best, and Baseline Methods

Table 1 shows all methods used in the 32 papers. Also, we provide an abbreviation of their names. We found some variations of Kernel Density Estimation (KDE), Autoregressive Models (AR), and Risk Terrain Modelling (RTM) that are also shown in column “Abbreviation” of table 1. In Table 4 of the paper, these variations are grouped to the “vanilla” methods and counted as KDE-based, AR-based, and RTM-based. In table 2, we show for each paper the data items proposed method, best proposed method, and baseline method.

Table S1: Overview of methods used in the selected 32 papers.

| ***No*** | ***Method name*** | ***Abbreviation*** |
| --- | --- | --- |
| 1 | Aggregate Autoregressive Model | AAR / AR-based (paper) |
| 2 | Auto Regression Moving Average | ARMA |
| 3 | Autoregressive Integrated Moving Average | ARIMA |
| 4 | Autoregressive Model | AR / AR-based (paper) |
| 5 | Bayessian Vector Autoregression | BVAR / AR-based (paper) |
| 6 | Cubic Spline Interpolation | CSI |
| 7 | Cumulative Logistic | CumLog |
| 8 | Decision Tree | DT |
| 9 | Deep Neural Network | DNN |
| 10 | DeepCrime | DeepCrime |
| 11 | Dynamic Covariance Kernel Density Estimation | DCKDE / KDE-based (paper) |
| 12 | Dynamic Spatial Distribution Approach | DSDA |
| 13 | Empirical Discriminative Tensor Analysis | EDTA |
| 14 | Ensemble SVM, RF, LDA, DT | Ensemble A |
| 15 | Ensemble LogR, NN | Ensemble B |
| 16 | Ensemble SVM, MLP, NB | Ensemble C |
| 17 | Exponential Smoothing | ExpSmooth |
| 18 | Extremely Randomized Tree regressor | Extra-Tree |
| 19 | Four-Way DLA | FWDLA |
| 20 | Gated Recurrent Unit | GRU |
| 21 | Gradient-Boosting regressor | GB |
| 22 | Hawkes Point Process | HPP |
| 23 | Holt's Exponential Smoothing | H_ExpSmooth |
| 24 | K Nearest Neighbors | KNN |
| 25 | Kernel Density Estimation | KDE / KDE-based (paper) |
| 26 | LASSO | LASSO |
| 27 | Linear Discriminant Analysis | LDA |
| 28 | Linear Regression | LR |
| 29 | Logistic Regression | LogR |
| 30 | Log-Normal regression | LogNR |
| 31 | Long Short-Term Memory | LSTM |
| 32 | Marked Point Process | MPP |
| 33 | Memetic algorithm with Fuzzy Clustering | FCM |
| 34 | ML-EIS | ML-EIS |
| 35 | Moving Average | MA |
| 36 | MultiLayer Perceptron | MLP |
| 37 | Naive Bayes | NB |
| 38 | Naïve Differences | ND |
| 39 | Naïve Model | NM |
| 40 | Neural Network | NN |
| 41 | Network-Time Kernel Density Estimation | NTKDE / KDE-based (paper) |
| 42 | NNH Hotspot Analysis | NNH |
| 43 | Normal Regression (linear) | LR |
| 44 | Offline Tensor Analysis | OTA |
| 45 | Point Process | PP |
| 46 | Poisson Regression | PoissonR |
| 47 | Polynomial Regression | PR |
| 48 | Prospective Hotspot Mapping | ProMap |
| 49 | Random Forest | RF |
| 50 | Random Prediction | RP |
| 51 | Random Walk | RW / AR-based (paper) |
| 52 | Recurrent Neural Networks | RNN |
| 53 | Risk Terrain Modelling | RTM |
| 54 | Risk Terrain Modelling with Negative Binomial Regression | RTM_NBR / RTM (paper) |
| 55 | Risk Terrain Modelling with Poisson Regression | RTM_PoissonR/ RTM (paper) |
| 56 | Seasonal Trend decomposition based on Loess | STL |
| 57 | Simple Spatial Disaggregation Approach | SSDA |
| 58 | Space Time Autoregressive Model | STAR |
| 59 | Spatiotemporal Kernel Density Estimation | STKDE / KDE-based (paper) |
| 60 | Spatio-Temporal Multi-Task Learning | stMTL |
| 61 | Support Vector Machines | SVM |
| 62 | TCP | TCP |
| 63 | Tensor Decomposition | TriMine |
| 64 | Univariate Extrapolative Method | UEM |
| 65 | Vector Autoregression | VAR / AR-based (paper) |
| 66 | Wide and Deep Learning | Wide&Deep |

Table S2: Proposed method, best proposed method, and baseline method used in the selected 32 papers. **(No* =*Reference number of the paper)***

| ***No**** | ***Proposed method*** | ***Best proposed method*** | ***Baseline method*** |
| --- | --- | --- | --- |
| 1 | MLP | MLP | ARIMA |
| 2 | MLP, KNN, RF | MLP | None |
| 3 | RF | RF | RP |
| 4 | LN, LogNR, PoissionR, CumLog | LogNR | AR |
| 5 | LR, MLP | MLP | AR, UEM |
| 6 | PR, SVM | SVM | AR |
| 7 | RTM_NBR, NNH | RTM_NBR | LogR |
| 8 | RTM_NBR, RTM_PoissonR | RTM_NBR | None |
| 9 | RTM_NBR, RTM_PoissonR | RTM | None |
| 10 | ExpSmooth, H_ExpSmooth | H_ExpSmoothing | AR, RW, lag 12 |
| 11 | KDE | KDE | None |
| 12 | STKDE | STKDE | KDE, ProMap |
| 13 | DeepCrime | DeepCrime | ARIMA, SVM, LR, MLP, Wide&Deep, GRU, TriMine |
| 14 | DSDA | DSDA | SSDA |
| 15 | KDE | KDE | None |
| 16 | RF, Extra Tree, GB | GB, RF | None |
| 17 | ML-EIS | ML-EIS | ExpSmooth, PR |
| 18 | DNN, RF, SVM, KNN | DNN | MA |
| 19 | DCKDE | DCKDE | STL |
| 20 | MPP | MPP | PP |
| 21 | RF, LogR | RF, LogR | LogR |
| 22 | HPP | HPP | None |
| 23 | EDTA | EDTA | FWDLA, OTA |
| 24 | FCM | FCM | None |
| 25 | NTKDE | NTKDE | STKDE |
| 26 | Ensemble A, RF, NN, SVM, LogR | Ensemble A, RF | None |
| 27 | Ensemble B, LogR, MLP | MLP | None |
| 28 | STAR | STAR | AAR, VAR, BVAR, NM, ND |
| 29 | LogR, NB, SVM, MLP, DT, RF | RF | None |
| 30 | SVM, MLP, DT, NB, Ensemble C | MLP | KNN |
| 31 | TCP | TCP | stMTL, LR, LASSO, ARMA, CSI |
| 32 | LSTM, RNN, GRU | LSTM | DT, NB, RF, KNN, LogR, MLP |

## Additional file D: PRISMA Checklist

| **Section/topic** | **#** | **Checklist item** | **Reported on section #** |
| --- | --- | --- | --- |
| **TITLE** | | | |
| Title | 1 | Identify the report as a systematic review, meta-analysis, or both. | Title |
| **ABSTRACT** | | | |
| Structured summary | 2 | Provide a structured summary including, as applicable: background; objectives; data sources; study eligibility criteria, participants, and interventions; study appraisal and synthesis methods; results; limitations; conclusions and implications of key findings; systematic review registration number. | Abstract |
| **INTRODUCTION** | | | |
| Rationale | 3 | Describe the rationale for the review in the context of what is already known. | 1 & 2 |
| Objectives | 4 | Provide an explicit statement of questions being addressed with reference to participants, interventions, comparisons, outcomes, and study design (PICOS). | 1 |
| **METHODS** | | | |
| Protocol and registration | 5 | Indicate if a review protocol exists, if and where it can be accessed (e.g., Web address), and, if available, provide registration information including registration number. | 3.1 |
| Eligibility criteria | 6 | Specify study characteristics (e.g., PICOS, length of follow-up) and report characteristics (e.g., years considered, language, publication status) used as criteria for eligibility, giving rationale. | 3.1 |
| Information sources | 7 | Describe all information sources (e.g., databases with dates of coverage, contact with study authors to identify additional studies) in the search and date last searched. | 3.1 |
| Search | 8 | Present full electronic search strategy for at least one database, including any limits used, such that it could be repeated. | 3.1 |
| Study selection | 9 | State the process for selecting studies (i.e., screening, eligibility, included in systematic review, and, if applicable, included in the meta-analysis). | 3.1 |
| Data collection process | 10 | Describe method of data extraction from reports (e.g., piloted forms, independently, in duplicate) and any processes for obtaining and confirming data from investigators. | 3.2 |
| Data items | 11 | List and define all variables for which data were sought (e.g., PICOS, funding sources) and any assumptions and simplifications made. | 3.2 |
| Risk of bias in individual studies | 12 | Describe methods used for assessing risk of bias of individual studies (including specification of whether this was done at the study or outcome level), and how this information is to be used in any data synthesis. | 3.2 |
| Summary measures | 13 | State the principal summary measures (e.g., risk ratio, difference in means). | - |
| Synthesis of results | 14 | Describe the methods of handling data and combining results of studies, if done, including measures of consistency (e.g., I^2^) for each meta-analysis. | - |
| Risk of bias across studies | 15 | Specify any assessment of risk of bias that may affect the cumulative evidence (e.g., publication bias, selective reporting within studies). | 3.2 |
| Additional analyses | 16 | Describe methods of additional analyses (e.g., sensitivity or subgroup analyses, meta-regression), if done, indicating which were pre-specified. | - |
| **RESULTS** | | | |
| Study selection | 17 | Give numbers of studies screened, assessed for eligibility, and included in the review, with reasons for exclusions at each stage, ideally with a flow diagram. | 3.1 |
| Study characteristics | 18 | For each study, present characteristics for which data were extracted (e.g., study size, PICOS, follow-up period) and provide the citations. | 4.1, 4.2, 4.3, 4.4 |
| Risk of bias within studies | 19 | Present data on risk of bias of each study and, if available, any outcome level assessment (see item 12). | - |
| Results of individual studies | 20 | For all outcomes considered (benefits or harms), present, for each study: (a) simple summary data for each intervention group (b) effect estimates and confidence intervals, ideally with a forest plot. | - |
| Synthesis of results | 21 | Present results of each meta-analysis done, including confidence intervals and measures of consistency. | - |
| Risk of bias across studies | 22 | Present results of any assessment of risk of bias across studies (see Item 15). | 3.2 |
| Additional analysis | 23 | Give results of additional analyses, if done (e.g., sensitivity or subgroup analyses, meta-regression [see Item 16]). | - |
| **DISCUSSION** | | | |
| Summary of evidence | 24 | Summarize the main findings including the strength of evidence for each main outcome; consider their relevance to key groups (e.g., healthcare providers, users, and policy makers). | 5 |
| Limitations | 25 | Discuss limitations at study and outcome level (e.g., risk of bias), and at review-level (e.g., incomplete retrieval of identified research, reporting bias). | 5 |
| Conclusions | 26 | Provide a general interpretation of the results in the context of other evidence, and implications for future research. | 6 |
| **FUNDING** | | | |
| Funding | 27 | Describe sources of funding for the systematic review and other support (e.g., supply of data); role of funders for the systematic review. | Funding |
